# Spirituality as a Scientific Construct: Testing Its Universality across Cultures and Languages

**DOI:** 10.1371/journal.pone.0117701

**Published:** 2015-03-03

**Authors:** Douglas A. MacDonald, Harris L. Friedman, Jacek Brewczynski, Daniel Holland, Kiran Kumar K. Salagame, K. Krishna Mohan, Zuzana Ondriasova Gubrij, Hye Wook Cheong

**Affiliations:** 1 University of Detroit Mercy, Detroit, Michigan, United States of America; 2 University of Florida, Gainsville, Florida, United States of America; 3 Veteran Affairs and University of Utah, Salt Lake City, Utah, United States of America; 4 The Neurobehavior Center of Minnesota, Edina, Minnesota, United States of America; 5 University of Mysore and Indian Council of Social Sciences Research, New Delhi, Manasagangotri, Mysore, India; 6 Makerere University, Kampala, Uganda and Mahidol University, Bangkok, Thailand; 7 University of Arkansas for Medical Sciences, Little Rock, Arkansas, United States of America; 8 Dongwoo Fine-Chem Co., Ltd. Mental Health Center, Pyeong Taek-Si, Gyeonggi-Do, South Korea; Institut Pluridisciplinaire Hubert Curien, FRANCE

## Abstract

Using data obtained from 4004 participants across eight countries (Canada, India, Japan, Korea, Poland, Slovakia, Uganda, and the U.S.), the factorial reliability, validity and structural/measurement invariance of a 30-item version of Expressions of Spirituality Inventory (ESI-R) was evaluated. The ESI-R measures a five factor model of spirituality developed through the conjoint factor analysis of several extant measures of spiritual constructs. Exploratory factor analyses of pooled data provided evidence that the five ESI-R factors are reliable. Confirmatory analyses comparing four and five factor models revealed that the five dimensional model demonstrates superior goodness-of-fit with all cultural samples and suggest that the ESI-R may be viewed as structurally invariant. Measurement invariance, however, was not supported as manifested in significant differences in item and dimension scores and in significantly poorer fit when factor loadings were constrained to equality across all samples. Exploratory analyses with a second adjective measure of spirituality using American, Indian, and Ugandan samples identified three replicable factors which correlated with ESI-R dimensions in a manner supportive of convergent validity. The paper concludes with a discussion of the meaning of the findings and directions needed for future research.

## Introduction

Interest in spirituality has grown considerably in a variety of scientific and health disciplines including, but not limited to, psychology, medicine, nursing, social work, counseling, sociology, and organizational management. As a manifestation of this interest, significant efforts have been put forth to generate conceptualizations of the construct which make it accessible to quantitative research. However, despite such efforts, there exists a fair amount of divergence and disagreement regarding what does and does not constitute the content domain of spirituality [1]. In fact, the main points of debate can be organized around four inter-related issues.

The first concerns the extent to which spirituality can be treated as separate from religion and religiousness and defined in a way which does not invoke theistic and metaphysical concepts [2–10]. The second relates to the degree of complexity of the construct; while it appears investigators in the area generally concur that spirituality is multidimensional, there is little consensus regarding the number and content of the dimensions to be included to sufficiently and thoroughly delineate it [5, 11–13]. The third centers upon the emerging recognition that many definitions of spirituality, particularly those that try to operationalize it as separate from religion, may be contaminated with well-being concepts [2, 14–16]. The last involves whether or not spirituality can be understood as a universal domain of functioning rather then as something that is expressed in unique and specific ways across age, sex, and, most importantly for the present study, culture [17–20].

In regard to the fourth issue, as the social, behavioral, and health sciences have started to more energetically embrace multiculturalism and has come to view religion as an important aspect of cultural difference [21], attention to establishing the manner in which spirituality is an emic or etic construct has risen. This is an important development in our view, not just for studies of spirituality and religion, but for all of science since there are indications that empirical findings may not generalize outside of the cultural environments in which they are investigated [22, 23]. In this vein, a cursory survey of the available cross-cultural literature on spirituality provides a somewhat conflicted picture. On the one hand, investigations on quantitative tests such as the Spiritual Transcendence Scale (STS) [24], the Brief Multidimensional Measure of Religiousness/Spirituality (BMMRS) [25], the Daily Spiritual Experiences Scale [26], the Faith Maturity Scale [27], and the Religious Coping Questionnaire (RCOPE) [28] offer some indications that they demonstrate satisfactory reliability and reasonably good factorial, convergent, criterion and/or incremental validity with different cultural, ethnic, and religious groups [29–34]. However, at the same time, studies utilizing more qualitative modes of inquiry have tended to provide argumentation and evidence pointing to spirituality as being a culturally bound concept [35–38]. Given this state of affairs, it is difficult to discern whether or not the contradictory findings are the product of methodological biases or weaknesses or instead reflect something substantive about the nature of spirituality which may require a reconsideration of how it should be studied scientifically.

Notwithstanding the incongruence of findings across methodologies which deserves attention in its own right but is beyond the scope of this paper, a more critical inspection of the published psychometric research indicates that the evidence backing an etic view of spirituality is not without discrepancies and limitations. For example, as with all areas of quantitative research in psychology and the behavioral sciences, there are studies which produce contradictory results. To illustrate, with the Spiritual Transcendence Scale, which is perhaps the most extensively studied test with different cultural and ethnic groups, research done in Australia and the Czech Republic has failed to find strong support for Piedmont’s [24] factor structure [39, 40].

More generally, virtually all of the extant investigations show inadequacies in sampling and statistical analysis. In terms of the former, there is a lack of sampling across multiple cultures within a single study. Instead, the prevailing trend has been to use data drawn from one culture [1, 32, 33, 41] and for the researchers to make broad generalizations about cross-cultural validity based upon the properties of the measurement tool within that culture. With respect to the latter, the available empirical studies have yet to employ stringent structural and measurement invariance testing involving direct evaluation of model fit using appropriate statistical procedures across tenable alternative models [42–45]. Ostensibly, if anyone is to claim that a measure and its associated concepts are universally valid, then there is a need to not only incorporate samples from a variety of cultures to better ensure that results are actually generalizable but it is also important to be sure that the model of choice demonstrates superior fit to the data as compared to reasonable alternative models.

Another set of issues plaguing existing quantitative research concerns the measures themselves. Nearly all tests that have been the focus of study have been developed by American researchers from a predominantly Western Judeo-Christian perspective using American samples. As has been argued by some [38], concepts such as transcendence and faith that are the focus of many tests are often implicitly couched within Western philosophy and religious doctrine in a manner which makes their appropriateness for use in cross-cultural research dubious. Also, most instruments were devised either to measure fairly specific concepts or were designed for relatively specific purposes. For instance, the Brief Multidimensional Measure of Religiousness/Spirituality was constructed not with regard to what actually should or should not comprise spirituality but instead was created expressly for the purposes of identifying the types of spiritual and religious variables which appear to hold the most potential to uncover a relation with physical and psychological health. Though it may be presumed that empirical data supporting the validity of a specialized assessment tool across cultures provides some basis to think that tests of similar but more inclusive concepts will also show validity, this is really a matter of empirical verification and one which requires the utilization of instruments which are not delimited to representing only selected elements and features of spirituality but instead are more overtly aimed at inclusively identifying and incorporating all its major components and traits [46].

Taken together, it should be apparent that in order for any substantive advances in the cross-cultural study of spirituality to occur, effort must be made to address as many of the aforementioned problems as possible. With this in mind, the purpose of the present study was to investigate the validity and generalizability of spirituality as a quantitative construct across cultures and languages with attention given to these challenges.

### But What is Spirituality? The Need for an Adequate Definition and Taxonomy

As a logical starting point, it struck us as important to first overview some of the general definitions of spirituality that are reflective of the better scholarship in the area so as to identify potential commonalities on which to base our own conceptual and methodological approach to the topic. Curiously, and despite the points of controversy cited earlier, there appears to be a fair amount of consistency, with most definitions placing emphasis on transcendence as a core feature. For instance, Elkins, Hedstrom, Hughes, Leaf, and Saunders [47] define spirituality as “a way of being and experiencing that comes about through awareness of a transcendent dimension and that is characterized by certain identifiable values in regard to self, others, nature, life, and whatever one considers to be the Ultimate” (p.10). The Fetzer Institute/National Institute on Aging Working Group [25] characterize spirituality as being “concerned with the transcendent, addressing ultimate questions about life’s meaning, with the assumption that there is more to life than what we see or fully understand” (p. 2). More recently, Pargament [48] has defined spirituality as the “sacred domain” which concerns “ideas of God, higher powers, divinity, and transcendent reality” (p. 32). As well, Koenig [49] sees spirituality as “distinguished from all other things—humanism, values, morals, and mental health—by its connection to the sacred, the transcendent…Spirituality is intimately connected to the supernatural and religion, although it extends beyond religion” (p. 116–117). As a final example, de Jager Meezenbrock and colleagues [2] define spirituality as “one’s striving for an experience of connection with oneself, connectedness with others and nature and connectedness with the transcendent” (p. 338).

Based upon this small sampling of definitions which have appeared across four decades of research, one might conclude that the most efficient and straightforward way of construing spirituality is to define it as that aspect of human functioning, experience, and existence which concerns the transcendent. Admittedly, there is a certain appeal to such a parsimonious definition as it appears to reconcile areas of divergence across researchers. Unfortunately, it embodies a problem already alluded to, namely it utilizes the concept of transcendence and, more specifically, transcendent reality as the cornerstone for how to understand spirituality. As argued most poignantly by Helminiak [50–52] such a term is presumptively linked to religious and theological systems that themselves are concerned with what is essentially metaphysical/supernatural and do not fit well with the contemporary scientific worldview.

Though we very much appreciate Helminiak’s position on the matter, we do not precisely share it as we consider it possible to use the term transcendent in a manner which minimizes its other worldly and supernatural connotations as would be the case if it was explicitly employed as an adjective to convey the phenomenological qualities of spirituality (particularly spiritual experience) or, alternatively, if it were framed as a psychological process responsible for shifts in identity and personological functioning (e.g., see [53]). Nonetheless, we maintain that the use of such terminology runs the risk of fostering misunderstanding around the veridical nature of spirituality itself, as there does not appear to be any way within science of establishing whether or not such terms actually represent something “real,” which is not reducible to more recognized and accessible biopsychosocial processes and mechanisms, or only signify something that is just an aberration of psychological functioning. The extensive debates concerning the differentiation of spirituality and psychopathology (especially dissociation and psychosis) replete in the literature may be seen as a tangible product of the confusion caused by the attribution of certain experiential states to so-called transcendent dimensions which can accompany the use of such terms [54–62]. Consequently, while there is no doubt in our minds that transcendence and other related concepts belong to the domain of spirituality, such notions are probably not best used to delineate its cardinal nature definitionally, at least if the goal is to arrive at a scientifically defendable conceptualization of spirituality.

An alternative approach to defining spirituality is to place emphasis on it as a natural phenomenon that is most centrally experiential in nature but is also accompanied by and/or manifested in neurophysiological, cognitive, characterological, and behavioral expressions that can be viewed as antecedent to, concomitant with, or determined by experience. The experiential core of spirituality itself can be conceptualized as consisting of experiences that (a) have certain phenomenological qualities involving modifications in the operations of self and identity relative to normal modes of functioning which have brain based correlates [63–65], (b) have an impact on thought, behavior, lifestyle, and personality and (c) lead to lasting changes in how one understands self, other, and the universe [66].

Such a definitional approach is copasetic with the work of several researchers and theoreticians; for instance, Grof [67,68] has argued that spirituality is a natural human developmental potential which involves shifts and transformations in conventional personality functioning that result in higher modes of health, integration, and well-being. Also, Hay and Socha [69] have proffered that spirituality is a natural human phenomenon which has both evolutionary and sociocultural significance. Finally, through the concept of self-transcendence, Cloninger [70] has incorporated spirituality into his biopsychosocial model of temperament and character.

A major advantage of defining spirituality in this manner is that it permits for its inclusion within naturalistic science in a way which does not explicitly require the use of religious and theological ideas but, at the same time, does not completely deny the utilization of such ideas and systems of thought as hermeneutic tools for the interpretation of spiritual phenomena. It also opens up the possibility of exploration and investigation of practices such as prayer, meditation, and contemplation as vehicles for facilitating the activation and maintenance of spirituality in a manner that is not constrained to the confines of doctrinal or institutional religiosity. In other words, while not wholly synonymous with spirituality, religion can be regarded as a major agent for fostering the emergence and understanding of spiritual experience and its significance for self, other, and reality without stifling scientific inquiry [71].

Considering its benefits and especially how it helps to address the problems associated with the use of metaphysical concepts, we elected to use this approach for a definition of general spirituality. Succinctly stated, spirituality is a natural aspect of human functioning which relates to a special class of non-ordinary experiences and the beliefs, attitudes, and behaviors that cause, co-occur, and/or result from such experiences. The experiences themselves are characterized as involving states and modes of consciousness which alter the functions and expressions of self and personality and impact the way in which we perceive and understand ourselves, others, and reality as a whole.

With this working definition of spirituality, it should be apparent that spirituality is complex, involving experiences with specific phenomenological features as well as cognitive and behavioral components. The question now becomes—what are the unique qualities and dimensions that make up this complex domain of functioning? As noted earlier, there appears to be fairly broad agreement that spirituality is a multi-faceted concept. However, the number and content of those facets vary across the available models and measures with some proposing a few as two [72] and others as many as nine [47]. Such a state of affairs presents considerable difficulties for researchers as there is little by way of guidance as to which one would be most suitable to use as a comprehensive model for cross-cultural research. For the sake of the present study, we elected to use the model of MacDonald [12, 73].

### Expressions of Spirituality: Model and measure

Motivated to address the problems with definition and measurement seen in the research, MacDonald [12] completed a series of conjoint exploratory factor analyses using a wide variety of instruments designed to assess spirituality and related concepts available in the literature with data obtained from two large samples of Canadian university students. His findings provided strong evidence supporting the existence of five robust dimensions which he argued could serve as a framework for organizing existing empirical findings on the relation of spirituality to other aspects of functioning (e.g., health, personality, social behavior) and provide direction for future research and theory development. The dimensions are Cognitive Orientation toward Spirituality (i.e., beliefs about the existence, validity, and relevance of spirituality for one’s sense of identity and daily functioning), Experiential/ Phenomenological Dimension (i.e., spiritual, mystical, religious, and transcendent experience and their phenomenological features including changed sense of self and perceptions of sacredness, divinity, holiness, and connectedness commonly tied to such experiences), Existential Well-Being (i.e., sense of meaning and purpose and perceived capacity to handle the existential adversities of life), Paranormal Beliefs (i.e., beliefs in the existence of paranormal phenomena and abilities) and Religiousness (i.e., intrinsic commitment to religious ideas, values, and practice for their own sake). Concurrent to developing the model, MacDonald also constructed a 100-item measure to operationalize the five dimensions. Named the Expressions of Spirituality Inventory (ESI), MacDonald [12, 73] found the instrument to demonstrate satisfactory reliability and convergent, discriminant, criterion, and factorial validity. Immediately subsequent to the publication of his initial findings, MacDonald [73] devised a shorter 32-item version of the test (ESI-Revised or ESI-R) using items from the parent scale selected on the basis of the uniqueness of content and item-to-scale reliability.

MacDonald’s [12] model and measure have a variety of strengths which make them ideal for our purposes. First, the model appears to capture the common latent constructs that are tapped by available spirituality instruments in a way which makes it one of the most comprehensive approaches to spirituality presently available. As a concrete illustration of this, MacDonald [12] found in his analyses that all dimensions were comprised of strong loadings from two or more measures but none of the instruments employed in his analyses, including those that are themselves attempts at comprehensive models [47, 74], loaded substantively on all five dimensions. With this mind, it is important to acknowledge that some investigators have been critical about the inclusion of paranormal beliefs in his analyses and model [75], and that MacDonald himself has raised issues with existential well-being representing a discrete aspect of spirituality [15, 76]. In response to such criticisms, particularly the latter one, it is important to remember that the dimensional model is based upon the latent constructs found within existing tests. In regard to the former criticism, MacDonald [12] has justified the incorporation of paranormal beliefs by pointing out that many faith systems accommodate beliefs in phenomena typically considered paranormal (e.g., miracles in the Judeo-Christian-Islamic traditions, siddhis in the Hindu tradition). More recently, independent research has suggested that spirituality may be best differentiated from other concepts through belief in supernatural spirits [77]. MacDonald found that such beliefs contribute to his paranormal beliefs dimension. Thus, it may be argued that paranormal beliefs have a place within a comprehensive model of spirituality.

A second asset of MacDonald’s model relates to his efforts at addressing the problems linked to defining spirituality as separate from religion. In particular, he was cognizant of the issues associated with the relation of spirituality to religion and to the predominance of Western conceptualizations of spirituality in the psychological literature. In response, when planning his analyses, he made sure to include tests that measure constructs derived from Eastern cultural and faith systems [78, 79] as well as instruments tapping religious variables most commonly associated with devout belief and practice thought to represent spirituality (i.e., intrinsic religiousness) [14, 80, 81]. The result was the identification of a dimension (i.e., Religiousness) which incorporates religious practice and beliefs in the existence of a higher power but that excludes doctrinal religiosity. Though his factor analytic findings suggest that the religiousness factor is more reflective of Western religious traditions (e.g., a measure of Eastern spirituality loaded negatively while a measure of Western spirituality loaded positively), statistical comparisons of people with various religious backgrounds on this dimension did not uncover any significant differences between denominational groups [12].

As a third strength, the model and/or instrument have been used in a variety of studies and has proven useful for theory development [71], test validation [82, 83], and empirical investigations of the relation of spirituality to personality, social, and health variables [9, 84–89]. As a product of this work, the model has also helped to clarify how spirituality manifests multifarious relations to functioning with some dimensions showing more positive associations (e.g., Existential Well-Being, Cognitive Orientation, and Religiousness), and others showing mixed (e.g. Experiential/Phenomenological Dimension) to negative relations (Paranormal Beliefs) [90, 91]. Thus, each dimension appears to uniquely and incrementally contribute to our understanding of how spirituality impacts functioning.

A fourth advantage, which may appear on the surface to be a liability, concerns the pattern of intercorrelations between the dimensions. MacDonald [12] found significant correlations between various pairs of the five ESI dimensions with the association between Religiousness and Cognitive Orientation emerging as conspicuously high (e.g., when correlating factor scores for these dimensions, r = .63; when correlating ESI dimension scores, r = .73). Though his factor analytic work revealed that these dimensions emerged separately, it may be argued that such results were the product of the samples used and that a four dimensional model wherein Religiousness and Cognitive Orientation are combined would be more parsimonious. This provides a good basis on which to compare and test four and five factor models to determine the best fitting model.

Fifth and finally, the ESI is one of the few measures of spirituality to include items to assess response validity (i.e., honesty of responding) and, as importantly, face validity (i.e., the respondent’s perception that the test is actually measuring spirituality). Since spirituality is a highly subjective phenomenon which could be said to be essentially ineffable [7], it would be unreasonable to expect any test to wholly and accurately capture it as it is directly known within a person’s experience. With the inclusion of a face validity item, researchers are permitted the opportunity to directly evaluate the extent to which different test-takers see the test as measuring something that is similar to their own understanding of spirituality.

### Study Design and Hypotheses

In order to provide a rigorous evaluation of the cross-cultural generalizability of spirituality as a psychometric construct, we adopted a complex approach to study design that attempted to address to the various shortcomings of the available research. In particular, we aimed to examine the reliability, factorial validity, and configural and measurement invariance of the ESI-R across samples drawn from several countries in disparate geographic areas that included both individualistic and collectivistic cultures. To assess the impact of culture alone versus culture and language together, samples from cultures in which English is a dominant language received all measures in English while the ESI-R and other tools used were translated to the dominant language spoken for the remaining samples. To ensure that our findings were not merely the product of the ESI-R, we incorporated a second novel questionnaire of spirituality developed through a content analysis of narrative descriptions of a spiritual person (see method section) to determine if it produced a factor structure akin to that of the ESI-R and to serve as a convergent validation measure.

In terms of research expectations, several hypotheses were tested. In particular, for all cultural samples, it was expected that (a) the ESI-R would be perceived as measuring spirituality as per responses to the face validity item, (b) the ESI-R would demonstrate satisfactory reliability and similar patterns of intercorrelations between dimensions as well as associations with demographic variables (i.e., age and sex), (c) the ESI-R would show structural or configural invariance with the original five factor model demonstrating superior goodness of fit to the data relative to a four factor model in which COS and REL dimensions are combined into a single dimension, (d) the ESI-R would be found to demonstrate measurement invariance, and (e) the spirituality adjective measure would produce factors similar to the ESI-R which would also show a congruent pattern of intercorrelations with each other and associations with demographic variables.

## Methods

### Participants

Data used in the present study were obtained from 4325 university student volunteers across eight different countries including Canada (n = 938), India (n = 800), Japan (n = 205), Poland (n = 400), the Slovak Republic (n = 178), South Korea (n = 660), Uganda (n = 518), and the United States (n = 626). The Canadian data were originally used in MacDonald [12] but were included here to permit reanalysis and comparison to the other samples. The remaining data were gathered between 2000 and 2006. Data for the Polish sample were obtained as part of a study on religious orientation but were not used in that study [92]. Finally, the data of 247 participants of the American sample have been used in a study to examine the relation of spirituality to well-being measures [15].

### Measures

#### Demographic Survey

A survey form was used which obtained basic demographic information (e.g., age, sex, religious affiliation).

#### Expressions of Spirituality Inventory-Revised (ESI-R) [73]

The ESI-R is a 32-item self-report questionnaire developed from a longer 100-item parent instrument [12, 76] designed to operationalize a five dimensional model of spirituality created through the conjoint factor analysis of 19 different tests selected due to their perceived representativeness of the content domain of spirituality. While the items for the 100-item ESI were included in the test on the basis of factor and reliability analyses, the selection of 30 of the items for the ESI-R was based upon item content uniqueness and reliability (e.g., corrected item-to-scale total correlations). The last two items are the same as the parent version of the test; item 31 is a face validity item (“This test appears to be measuring spirituality”) and item 32 an honesty-of-responding item (“I have responded to all items honestly”). Each dimension is tapped by six items. The test employs a five point scale ranging from 0 (Strongly Disagree) to 4 (Strongly agree) which is used by respondents to rate the extent to which they agree with the content of the item. For the interested reader, the 30 items for the test appear in a table in the results section.

#### Spirituality Adjective List (SAL)[93]

The SAL is a 40 item self-report instrument that utilizes a five point response scale ranging from 0 (Strongly Disagree) to 4 (Strongly Agree). The items were developed from a thematic content analysis of written narrative descriptions of a spiritual person obtained from 50 Canadian university students. In completing the analysis, the test authors first reviewed the written narratives and identified clear descriptors/adjectives. Thereafter, the authors devised items to embody adjectives that were found to be most commonly used across respondents. Items for the SAL in order of appearance on the questionaire are as follows: 1) I have strong beliefs and convictions, 2) I am devoted to what I believe in, 3) I am at peace with others, 4) I am moral, 5) I understand myself, 6) I meditate, 7) I am holy, 8) I am religious, 9) I pray, 10) I believe in a higher power, 11) I am at peace with myself, 12) I am happy, 13) I believe in spirits or ghosts, 14) I am concerned about the meaning of life, 15) I am a positive person, 16) I believe in life after death, 17) I feel a sense of wholeness or completeness, 18) I am in touch with my soul, 19) I appreciate nature, 20) I help others, 21) I take time to reflect on who I am, 22) I engage in private activities which help me feel a sense of connection with a higher power, 23) I attend church services, 24) I believe in supernatural powers, 25) I have a sense of meaning in life, 26) I am optimistic, 27) I believe that my soul will live on after I die, 28) I believe that the mind, body and soul are one, 29) I have a deep respect for all living things, 30) I understand myself spiritually, 31) I have a strong faith, 32) I am dedicated to my beliefs, 33) I am a caring person, 34) I am gentle, 35) I am an honest person, 36) I am in touch with my innermost thought, 37) I believe in God, 38) I am calm, 39) I am content, and 40) I am blessed.

### Procedure

The questionnaires were administered to students at universities in their respective countries. In all cases, brief presentations about the study and the need for participants were made to classes by one of the researchers and/or a research assistant under the supervision of one of the researchers. Students who expressed interest in participating completed paper-and-pencil copies of the measures either during class time, during scheduled testing sessions, or were given hardcopies to complete and return to the researcher or research assistant. In Canada, the United States, India, and Uganda, the instrument was given in English. For all the remaining samples save Poland, the test was translated using the standard translation-back translation procedure. For the Polish sample, the test was translated using a committee approach wherein a number of people fluent in both English and Polish collaboratively worked to create the translation [92]. Along with the ESI-R, the SAL was also given to respondents in the Indian, and Ugandan samples and to approximately 300 of the American participants.

### Ethics Statement

All data gathering was completed in a manner consistent with standard ethical practices for questionnaire based psychometric research in place at the time of data collection. Approval was obtained prior to data collection either through established institutional research review committees/boards or through institutional officials when such committees did not exist. For the American data, approval was granted by the University of Detroit Mercy Institutional Review Board. For the Canadian data, approval was obtained from the University of Windsor Research Ethics Committee. For both samples, the first author (D.A.M.) was the primary researcher involved with data collection. For the Indian data, permission was obtained from chairpersons and administrative heads of the University of Mysore Faculties of Arts and Humanities, Commence and Management, Education, Law, and Science and Technology. Data collection was completed by the fifth author (K.K.K.S.). For the Japanese data, approval was granted by the Academy of Counseling Japan and the Saybrook University Institutional Review Board. Data gathering was done by research assistants supervised by the second author (H.L.F). For the Korean data, approval was obtained from the Seoul Graduate School of Counseling Psychology and Buddhism research committee. Data collection was done by the eighth author (H.W.C.). For the Polish data, approval was obtained from the Dean of Studies at Kardynał Stafan Wyszyński University. Data collection was done by the third author (J.B.). For the Slovakian data, approval was obtained from the University of Arkansas at Little Rock Institutional Review Board and the University Vice Dean at Comenius Medical University. Data collection was completed by a research assistant under the supervision of the fourth (D. H.) and seventh authors (Z.O.G.). Finally, for the Ugandan data, approval was obtained from the Makerere University Institute of Psychology Higher Education Research Board. Data collection was completed by the sixth author (K. K. M.).

For the American, Canadian, Indian, Japanese, Korean, and Ugandan samples, written informed consent was obtained prior to the completion of the questionnaires. For the Polish and Slovakian samples, informed consent was communicated verbally. In these instances, only individuals who gave verbal consent were provided with hardcopies of the questionnaires to complete. Verbal consent was considered sufficient by the boards and/or officials at all involved institutions for questionnaire based psychometric research. For all samples, participation was voluntary and no personal identifying information was obtained.

### Data Analysis

The approach to analyzing data for this study was multi-tiered and involved looking at questionnaire scores at both the item and scale level and with the samples combined and separated. First, to ascertain the extent to which the ESI-R demonstrated face validity, responses to item 31 which asked participants to rate the extent to which they viewed the test as measuring spirituality were analyzed via ANOVA and examination of response frequencies. Next, descriptive statistics and reliabilities for the ESI-R items and dimension scores were calculated for all samples combined and then for each country sample separately. ESI-R scores at both item and scale levels were then examined as a function of country via ANOVA. These analyses were done in response to the recommendations of Byrne and Watkins [43] who suggested that examination of score differences across cultures should be included in any evaluation of measurement invariance. Since there is evidence that the ESI-R dimensions may differ as a function of age and sex of respondent [12, 18], product-moment correlations were next calculated with the ESI-R dimensions and these two participant variables across all country samples. Thereafter, inter-correlations between the ESI-R dimensions were computed. Next, both exploratory (EFA) and confirmatory factor analyses (CFA) were completed in order to assess the structural consistency and factor and measurement invariance of the ESI-R dimensions. Both approaches were employed in this study in response to issues raised regarding the use of confirmatory factor in the evaluation of personality inventories [94, 95]. In consideration of the fact that the original ESI was developed using an EFA approach very similar to that employed for creating measures of the Five Factor Model of personality [12, 76], these issues seemed to us to be applicable to this study. The utilization of CFA based techniques to assess structural and measurement invariance was done in a manner consistent with experts in the area of Structural Equation Modeling and CFA [42, 44, 45, 96]. Finally, the SAL was examined across American, Ugandan, and Indian samples using EFA to identify latent factors and to construct subscales based upon similar patterns of varimax rotated factor loadings. Reliabilities of the emergent subscales, subscale intercorrelations, and correlations with the ESI dimensions were then calculated.

## Results

Prior to beginning any analyses, data were examined for completeness, accuracy, and evidence of response bias (e.g., perseverative responding, dishonest responding as indicated by a disagree response to item 32 on the ESI-R asking if questions were responded to honestly). Any cases demonstrating one or more of these problems were excluded from all analyses. This resulted in a total 321 participants being removed from the study. Table 1 presents information on the basic demographic characteristics for the total combined samples and for each country sample separately including age, gender, and religious affiliation.

**Table 1:** Description of Demographic Characteristics of Samples After Data Cleaning.

		Age		Sex		Religious Affiliation						
Sample	N	M	SD	F	M	C	J	I	H	B	O	N
Combined	4004	23.59	6.75	2558	1436	2167	11	115	615	120	187	789
Canada	932	20.97	4.33	673	259	716	6	26	11	9	56	108
India	718	22.29	1.79	385	330	46	0	31	603	16	2	20
Japan	182	34.67	9.99	131	46	5	0	0	0	31	11	135
Korea	560	28.37	9.11	326	234	40	0	0	0	62	110	348
Poland	395	21.57	1.90	292	103	381	0	1	0	1	1	11
Slovakia	178	22.15	6.89	166	11	128	0	0	0	1	0	49
Uganda	447	24.78	5.14	179	267	392	0	48	0	0	2	5
U.S.	592	22.10	7.07	406	186	459	5	9	1	0	5	113

*Note*. Where frequencies do not add up to the total sample size, data were missing. For religious affiliation, C = Christianity, J = Judaism, I = Islam, H = Hinduism, B = Buddhism, O = Other Religion, N = No Religion. No Religion includes both participants who self-identified as not having any religious affiliation and those who did not report anything to the item on religious affiliation.

### Face Validity of the ESI-R Across Country Samples

As an initial set of statistics, we focused on the response to item 31 that asks participants to rate the extent to which they perceive the ESI-R as measuring spirituality since it struck us as a good initial indicator of the extent to which spirituality in general and the instrument itself hold up across cultures. With all but three of the 4004 participants providing a response to this item, the mean response for all samples combined is 2.85 (SD = 1.06). Examination of frequencies for the pooled samples indicates that 70.4% of participants responded “agree” or “strongly agree” to the item. For a more detailed analysis, we examined differences across each country sample via a one-way ANOVA. Table 2 reports the descriptive statistics, response frequencies and ANOVA results. The ANOVA emerged significant (F(7, 3993) = 131.07, p<.001, η^2^ = .19). Post-hoc analyses (Scheffe’s test), uncovered a variety of statistically significant pair-wise differences between country samples. Notwithstanding these statistical findings, examination of response frequencies indicates that the majority of respondents from the Canadian, American, Indian, and Ugandan samples responded with agree or strongly agree to the item. For the remaining samples, the majority of responses fall between neutral to strongly agree.

**Table 2:** Descriptive and Frequency Statistics for ESI-R Item 31 Concerning Perception of Test as a Measure of Spirituality.

				Response Frequencies					
Country	N	M	S.D.	SD	D	N	A	SA	Sig Post-hoc
Total	4001	2.85	1.06	152	327	705	1615	1202	---
American(A)	592	3.29	0.90	8	25	55	204	300	I, P, J, S, K
Canadian(C)	932	3.19	0.87	11	37	106	387	391	I, P, J, S, K
Indian (I)	718	2.81	0.89	18	52	102	420	126	C, A, U, P, K
Japanese (J)	182	2.52	0.94	2	24	62	66	28	C, A, U, P, K
Korean (K)	560	2.10	1.20	70	89	196	128	77	C, A, I, U, J, S
Polish (P)	393	2.16	1.10	34	74	114	136	35	C, A, I, U, J, S
Slovakian (S)	177	2.79	0.98	5	11	44	73	44	C, A, U, P, K
Ugandan (U)	447	3.30	0.80	4	15	26	201	201	I, P, J, S, K

*Note*. Three respondents (two Polish and one Slovakian) did not provide a response to item 31 leaving a total combined sample of 4001. For response frequencies, SD = Strongly Disagree, D = Disagree, N = Neutral, A = Agree, SA = Strongly Agree. One-Way ANOVA as a function of country: F(7, 3993) = 131.07, p<.001, η^2^ = 0.19. Significant post-hoc pairwise differences based upon Scheffe test. All post-hoc findings significant at p<.05

In order to get a sense of the extent to which responses to the face validity item are associated with scores on the ESI-R, correlations were calculated between ESI-R item 31 and all ESI-R dimensions and items (35 correlations in all) using pooled data (N = 4001). With the dimension scores, all coefficients were significant at p<.01 or lower and ranged in absolute value from |.04| for EWB to |.17| for REL. For items, most correlations were small; nine coefficients fell at r = |.10| or higher with the strongest correlation falling at r = |.23|. Correlations were also examined for each country sample separately. For all countries, a similar pattern of coefficients was obtained with no correlation exceeding r = |.22|.

### Reliability Analyses of the ESI-R

Scale reliabilities were calculated for each ESI-R dimension for the total combined sample and each country sample separately. Tables 3 and 4 present the scale and item-level descriptive and reliability statistics for the total combined sample including Cronbach’s alphas and corrected item-to-scale total correlations for all items. Table 5 presents the scale level descriptive statistics and reliability coefficients for each country sample separately along with the mean corrected item-to-scale total correlations for all items within each ESI-R dimension.

**Table 3:** ESI-R Dimension Descriptive and Reliability Statistics for Combined Sample (N = 4004).

ESI-R Scale	Mean	S.D.	Alpha
Cognitive Orientation toward Spirituality (COS)	15.21	5.20	.89
Experiential/Phenomenological Dimension (EPD)	10.62	4.91	.81
Existential Well-Being (EWB)	15.05	4.30	.76
Paranormal Beliefs (PAR)	10.72	4.68	.72
Religiousness (REL)	14.64	5.85	.88

*Note*. Alpha = Cronbach’s Alpha

**Table 4:** ESI-R Item Descriptive and Reliability Statistics for Combined Sample (N = 4004).

ESI-R Item	Mean	S.D.	CISr
Cognitive Orientation toward Spirituality (COS) Items			
1. Spirituality is an important part of who I am as a person	2.69	1.09	.71
6. Spirituality is an essential part of human existence	2.71	1.07	.73
11. I am more aware of my lifestyle choices because of my spirituality	2.28	1.12	.69
16. I try to consider all elements of a problem, including its spiritual aspects, before I make a decision	2.27	1.08	.61
21. My life has benefited from my spirituality	2.53	1.12	.75
26. I believe that attention to one’s spiritual growth is important	2.74	1.01	.74
Experiential/Phenomenological Dimension (EPD) Items			
2. I have had an experience in which I seemed to be deeply connected to everything	2.02	1.08	.53
7. I have had an experience in which I seemed to transcend space and time	1.46	1.19	.54
12. I have had a mystical experience	1.67	1.17	.57
17. I have had an experience in which I seemed to merge with a power or force greater than myself	1.77	1.17	.63
22. I have had an experience in which all things seemed divine	1.77	1.10	.57
27. I have had an experience in which I seemed to go beyond my normal everyday sense of self	1.93	1.11	.62
Existential Well-Being (EWB) Items			
3. It always seems that I am doing things wrong (-)	2.73	1.00	.49
8. I am not comfortable with myself (-)	2.65	1.12	.52
13. Much of what I do in life seems strained (-)	2.31	1.08	.42
18. My life is often troublesome (-)	2.30	1.12	.59
23. I often feel tense (-)	1.92	1.09	.49
28. I am an unhappy person (-)	3.13	0.94	.50
Paranormal Beliefs (PAR) Items			
4. It is possible to communicate with the dead	1.50	1.27	.50
9. I believe witchcraft is real	1.49	1.23	.44
14. It is possible to predict the future	1.89	1.16	.46
19. I do not believe in spirits or ghosts (-)	2.29	1.25	.36
24. I think psychokinesis, or moving objects with one’s mind, is possible	1.80	1.17	.44
29. It is possible to leave your body	1.75	1.20	.48
Religiousness (REL) Items			
5. I believe that going to religious services is important	2.43	1.25	.63
10. I feel a sense of closeness to a higher power	2.32	1.17	.66
15. I see myself as a religiously oriented person	2.14	1.21	.73
20. I see God or a Higher power present in all the things I do	2.33	1.26	.70
25. I practice some form of prayer	2.58	1.23	.68
30. I believe that God or a Higher Power is responsible for my existence	2.83	1.31	.70

*Note*. CISr = Corrected item-to-scale total correlation. Numbers accompanying ESI-R item content reflects item number on test. (-) = Reverse worded and scored. ESI-R Items reprinted with permission of the test author.

**Table 5:** Descriptive and Reliability Statistics and One-way ANOVA results for ESI-R Dimensions as a Function of Country.

	ESI-R Dimension				
	COS	EPD	EWB	PAR	REL
American (A) (n = 592)					
Mean	15.56	10.60	16.22	10.85	16.41
Standard Deviation	5.35	4.93	4.36	4.59	5.19
Alpha	.92	.85	.84	.77	.89
Mean Corrected Item-Scale Correlation	.77	.63	.62	.52	.71
Canadian (C) (n = 932)					
Mean	14.41	9.90	14.93	12.47	13.65
Standard Deviation	4.95	4.76	4.53	5.15	5.80
Alpha	.87	.81	.80	.82	.89
Mean Corrected Item-Scale Correlation	.68	.57	.56	.59	.71
Indian (I) (n = 718)					
Mean	14.55	11.13	14.99	9.20	15.16
Standard Deviation	4.42	3.94	4.13	3.93	4.69
Alpha	.82	.71	.72	.59	.78
Mean Corrected Item-Scale Correlation	.59	.45	.46	.33	.53
Japanese (J) (n = 182)					
Mean	17.15	10.50	13.30	12.29	9.35
Standard Deviation	3.37	5.59	3.92	4.14	4.53
Alpha	.77	.89	.74	.79	.81
Mean Corrected Item-Scale Correlation	.51	.71	.49	.55	.57
Korean (K) (n = 560)					
Mean	12.41	9.49	15.30	8.10	10.66
Standard Deviation	6.10	6.02	4.14	4.31	6.21
Alpha	.91	.89	.78	.74	.89
Mean Corrected Item-Scale Correlation	.76	.72	.53	.47	.71
Polish (P) (n = 395)					
Mean	19.10	11.21	14.34	11.18	17.71
Standard Deviation	3.75	5.21	4.42	4.18	4.26
Alpha	.84	.80	.78	.65	.81
Mean Corrected Item-Scale Correlation	.63	.56	.53	.39	.58
Slovakian (S) (n = 178)					
Mean	12.93	9.80	13.74	12.22	11.65
Standard Deviation	4.46	3.95	3.70	3.93	5.36
Alpha	.86	.72	.70	.66	.85
Mean Corrected Item-Scale Correlation	.64	.46	.44	.39	.63
Ugandan (U) (n = 447)					
Mean	17.69	12.61	15.39	11.00	19.13
Standard Deviation	3.94	4.03	3.91	3.97	3.42
Alpha	.83	.70	.67	.45	.72
Mean Corrected Item-Scale Correlation	.61	.43	.40	.22	.45
One-Way ANOVA					
F (7, 3996)	97.03	20.96	15.74	68.02	168.86
η^2^	.15	.04	.03	.11	.23

*Note*. COS = Cognitive Orientation toward Spirituality, EPD = Experiential/ Phenomenological Dimension, EWB = Existential Well-Being, PAR = Paranormal Beliefs, REL = Religiousness. All ANOVA results significant at p<.001.

As can be seen in the tables, alphas and item-to-scale correlations are reasonably good for the total combined sample and each country sample with only a two notable exceptions. For the Indian and Ugandan samples, Paranormal Beliefs produced unacceptably low alpha coefficients (α = .59 and .45, respectively).

### Tests of Differences Across ESI-R Dimension Scores

A series of one-way Analyses of Variance (ANOVAs) were completed to see if significant differences exist on ESI-R dimension scores as a function of country (see Table 5). All five ANOVAs emerged significant (COS- F(7, 3996) = 97.03, p<.001; EPD- F(7, 3996) = 20.96, p<.001; EWB- F(7,3996) = 15.74, p<.001; PAR- F(7, 3996) = 68.02, p<.001; REL = F(7, 3996) = 168.86, p<.001). Effect sizes as reflected in eta-squared are small for EPD and EWB and medium to large for the remaining dimensions. Post hoc analyses (Scheffe’s test) revealed a large array of statistically significant (p<.05) pairwise differences for all five ANOVAs across the country samples. Only general trends in these findings will be described here. For COS, only two post-hoc tests were not significant (between Canada and India, and Slovakia and Korea). Similarly, for REL, only two pairwise comparisons, between Japan and Korea, and Slovakia and Korea, emerged non-significant. For EPD, Uganda was found to produce significantly higher scores than all other countries. The Polish and Indian samples produced significantly higher scores than Canada, Korea, and Slovakia. Last, the American sample has a significantly higher score compared to the Korean sample. For EWB, the American sample generated a significantly higher score than all other countries. At the other extreme, the Polish, Japanese, and Slovakian samples did not produce any pairwise differences with any countries. Finally, for PAR, the Indian and Korean samples produced significantly lower scores compared to each other and all other countries. The Canadian sample generated a significantly higher score than all other countries save Japan and Slovakia.

### Tests of Differences Across ESI-R Item Scores

One-way ANOVAs examining all 30 ESI-R item scores as a function of culture were calculated (see Table 6). Akin to what was found with the dimension scores, all 30 ANOVAs were significant with effect sizes ranging from small for all EPD items, small to medium for EWB items, and medium to large for COS, PAR, and REL items. Post-hoc analyses (Scheffe test) identified an extensive array of pairwise differences across the country samples for all items going from a minimum of three for item 3 (EWB) to 24 for items 20 (REL), 25 (REL), 26 (COS), and 30 (REL).

**Table 6:** One-way ANOVA Results for ESI-R Items as a Function of Country.

			Country							
ESI-R Item	F (7,3996)	η^2^	A	C	I	J	K	P	S	U
COS										
Item 1	56.75	.09	2.57	2.50	2.48	2.86	2.66	3.46	2.17	3.07
Item 6	100.46	.15	2.73	2.55	2.57	3.16	2.14	3.57	2.26	3.17
Item 11	53.94	.09	2.40	2.18	2.15	2.58	1.79	2.99	1.92	2.57
Item 16	48.58	.08	2.30	2.06	2.36	2.74	1.74	2.63	2.19	2.68
Item 21	62.12	.10	2.72	2.51	2.42	2.66	1.89	2.97	2.11	3.04
Item 26	92.86	.14	2.84	2.61	2.57	3.15	2.19	3.47	2.28	3.17
EPD										
Item 2	29.56	.05	2.03	1.74	2.10	2.09	2.04	2.59	1.67	2.02
Item 7	21.94	.04	1.34	1.24	1.73	1.43	1.27	1.57	1.34	1.87
Item 12	13.07	.02	1.59	1.67	1.62	1.92	1.46	1.53	1.89	2.04
Item 17	32.04	.05	1.90	1.56	1.92	1.76	1.45	1.77	1.49	2.36
Item 22	6.78	.01	1.74	1.75	1.87	1.46	1.65	1.88	1.58	1.93
Item 27	18.53	.03	2.01	1.94	1.89	1.84	1.63	1.86	1.81	2.39
EWB										
Item 3	8.73	.02	2.79	2.61	2.66	2.70	2.99	2.78	2.80	2.66
Item 8	41.25	.07	2.97	2.69	2.81	1.74	2.28	2.60	2.44	2.88
Item 13	81.32	.12	2.47	2.44	2.27	2.33	2.76	1.29	1.96	2.37
Item 18	15.65	.03	2.57	2.14	2.30	2.05	2.41	2.34	1.83	2.42
Item 23	20.82	.04	2.14	1.95	1.97	1.28	1.64	2.09	1.76	1.99
Item 28	7.98	.01	3.28	3.12	2.98	3.19	3.23	3.24	2.94	3.06
PAR										
Item 4	132.25	.19	1.68	1.99	1.03	1.58	0.54	2.27	1.96	1.30
Item 9	41.17	.07	1.43	1.81	1.04	1.71	1.58	1.00	1.68	1.81
Item 14	69.16	.11	1.93	2.24	1.86	2.21	1.13	1.51	2.30	2.17
Item 19	65.69	.10	2.51	2.59	1.70	2.46	2.44	2.79	2.13	1.69
Item 24	14.60	.02	1.56	1.89	1.98	2.15	1.61	1.61	2.08	1.84
Item 29	78.84	.12	1.74	1.94	1.60	2.18	0.82	2.00	2.08	2.20
REL										
Item 5	123.11	.18	2.57	2.06	2.37	2.02	1.94	3.18	1.80	3.49
Item 10	42.10	.07	2.60	2.29	2.40	1.85	1.86	2.23	1.96	2.87
Item 15	96.35	.14	2.36	1.88	2.09	1.33	1.58	2.87	2.04	2.87
Item 20	90.49	.14	2.40	2.10	2.52	1.29	1.96	2.85	1.61	3.17
Item 25	135.23	.19	3.03	2.51	2.78	1.24	1.79	3.23	1.98	3.06
Item 30	223.00	.28	3.45	2.81	3.01	1.62	1.54	3.35	2.27	3.67

*Note*. All ANOVAs significant at p<.001. COS = Cognitive Orientation toward Spirituality, EPD = Experiential/Phenomenological Dimension, EWB = Existential Well-Being, PAR = Paranormal Beliefs, REL = Religiousness For country, A = American, C = Canadian, I = Indian, J = Japanese, K = Korean, P = Polish, S = Slovakian, U = Ugandan.

### Associations of ESI-R Dimensions with Age and Sex

Product-moment correlations were computed between the ESI-R dimensions for age (in years) and sex (male coded 0 and female coded 1) for the total combined sample and each country sample separately (see Table 7). When considering the findings for the combined sample, while there are a number of statistically significant correlations, the coefficients are generally low in magnitude for both age and sex. Closer inspection of the findings across the country samples reveals some notable differences in the strength of associations. With age, the Korean sample followed by the Slovakian sample produced at least one correlation of moderate magnitude. With sex, the most substantive correlations were found with the American, Canadian, and Ugandan samples. Looking at the findings across the ESI-R dimensions, Religiousness and Cognitive Orientation toward Spirituality appear to be most strongly and significantly related to age and sex.

**Table 7:** Product-Moment Correlations Between ESI-R Dimensions and Demographic Variables.

	ESI-R Dimensions				
	COS	EPD	EWB	PAR	REL
Combined					
Age	.15***	.13***	.04**	.00	−.04*
Sex	.10***	−.03	.01	.07***	.08***
American					
Age	.16***	.12**	.18***	.05	.13**
Sex	.18***	.06	.06	.13**	.19***
Canadian					
Age	.14***	.10**	.04	−.03	.06
Sex	.19***	.06	−.02	.17***	.13***
Indian					
Age	.05	.09*	−.06	.09*	−.09*
Sex	.06	−.10**	.04	−.12**	.23***
Korean					
Age	.40***	.38***	.16***	.28***	.22***
Sex	.07	−.01	.05	.03	.12**
Japanese					
Age	.21**	.11	.08	−.00	.26**
Sex	.12	.06	.05	.17*	.12
Polish					
Age	.16**	.15**	−.08	.14**	.20***
Sex	.06	−.03	.02	−.11*	.04
Slovakian					
Age	.31***	.07	.09	−.02	.04
Sex	.01	.08	−.13	−.01	.19**
Ugandan					
Age	.05	−.01	−.02	.07	.07
Sex	.22***	−.03	.06	−.19***	.18***

*Note*. COS = Cognitive Orientation toward Spirituality, EPD = Experiential/Phenomenological Dimension, EWB = Existential Well-Being, PAR = Paranormal Beliefs, REL = Religiousness. For sex, male coded 0, female coded 1.

*p<.05

**p<.01

***p<.001

Given the significant findings obtained with age and sex and the observed significant differences for all ESI-R dimensions as a function of culture, it was surmised that interaction effects between the three variables should be examined. In response, five 2 (sex) x 2 (age; two groups based on pooled median split—18–21 years versus 22 and older) x 8 (country samples) ANOVAs were computed wherein each ESI-R dimension served as the dependent variable. In all five ANOVAs, non-significant three-way interactions and non-significant two-way interactions between sex and age were produced. For Existential Well-Being, no two-way interactions were significant. For Cognitive Orientation toward Spirituality, significant interactions between age and country (F(7, 3896) = 9.01, p<.001) and sex and country (F(7, 3896) = 3.78, p<.001) were found. A similar pattern of significant two-way interactions was generated for the Experiential/Phenomenological Dimension (for age x country- F(7, 3896) = 4.84, p<.001; for sex x country- F(7, 3896) = 2.02, p<.05). For Paranormal Beliefs, a significant interaction was obtained between sex and country (F(7, 3896) = 6.56, p<.001). Finally for Religiousness, a significant interaction was found between age and country (F(7, 3896) = 5.71, p<.001). Effect sizes for all significant interactions were small (eta-squared ranged from .00 to .02).

When running the 2x2x8 ANOVAs, examination of cell frequencies revealed that the Slovakian and Japanese samples had far too few participants in some subgroups. Since unequal cell sizes can have a distorting effect on ANOVA results, we re-ran all five analyses excluding the Slovakian and Japanese samples to make sure that evidence of interaction effects was robust. For COS, significant two-way interactions were found with sex and country (F(5, 3553) = 4.99, p<.001) and age and country (F(5, 3553) = 11.89, p<.001). For EPD, significant two-way interactions emerged for sex and country (F(5, 3553) = 2.44, p<.05) and age and country (F(5, 3553) = 6.32, p<.001). For EWB, significant two-way interactions were found for sex and age (F(1, 3553) = 3.88, p<.05) and age and country (F(5, 3553) = 2.70, p<.05). For PAR, a significant two way interaction emerged with sex and country (F(5, 3553) = 9.01, p<.001). Finally, for REL, a significant two-way interaction was found between age and country (F(5, 3553) = 7.14, p<.05) and a significant three-way interaction emerged (F(5, 3553) = 2.24, p<.05). While these results are not identical to those found using all country samples, thus suggesting that asymmetry of cell sizes did have some impact on our main analyses, they still point to the existence of interaction effects.

### Inter-correlations of ESI-R Dimensions

Product-moment correlations were next calculated between the ESI dimensions for pooled samples and for each country sample (see Table 8). Examination of these coefficients reveals a few conspicuous trends. In particular, the correlations between COS-REL, COS-EPD, EPD-REL, and EPD-PAR came out significant and of moderate to high strength in every set of analyses regardless of sample. Another observable trend concerns the three samples with the lowest numbers of Christian participants (i.e., Indian, Korean, and Japanese). Specifically, with these three samples, correlations of moderate strength were found between PAR-REL and PAR-COS. Lastly, though some statistically significant coefficients were obtained with EWB, the size of the coefficients for the total pooled sample and each country sample is consistently small.

**Table 8:** Inter-correlations of ESI-R Dimensions for Total Combined and Country Samples.

		ESI Dimension			
Country	ESI Dimension	COS	EPD	EWB	PAR
Combined	EPD	.48***			
	EWB	.04	−.04		
	PAR	.21***	.37***	−.09***	
	REL	.71***	.39***	.07***	.13***
American	EPD	.49***			
	EWB	.16***	.01		
	PAR	.04	.30***	−.09*	
	REL	.84***	.40***	.14***	.04
Canadian	EPD	.36***			
	EWB	.06	−.03		
	PAR	.14***	.36***	−.05	
	REL	.70***	.19***	.03	.04
Indian	EPD	.50***			
	EWB	−.06	−.13**		
	PAR	.33***	.37***	−.11**	
	REL	.66***	.40***	.01	.24***
Japanese	EPD	.39***			
	EWB	.02	.14		
	PAR	.20**	.52***	.18*	
	REL	.45***	.60***	.06	.44***
Korean	EPD	.70***			
	EWB	.02	−.00		
	PAR	.53***	.69***	−.14**	
	REL	.75***	.50***	−.02	.39***
Polish	EPD	.31***			
	EWB	.03	−.12*		
	PAR	−.02	.34***	−.13*	
	REL	.74***	.27***	.03	.05
Slovakian	EPD	.47***			
	EWB	−.02	−.19*		
	PAR	−.04	.23**	−.14	
	REL	.67***	.39***	−.08	−.10
Ugandan	EPD	.36***			
	EWB	.22***	−.08		
	PAR	−.07	.23***	−.10*	
	REL	.68***	.39***	.16**	−.06

*Note*.

*p<.05

**p<.01

***p<.001.

COS = Cognitive Orientation toward Spirituality, EPD = Experiential/Phenomenological Dimension, EWB = Existential Well-Being, PAR = Paranormal Beliefs, REL = Religiousness

### Exploratory Factor Analyses (EFAs)

Since the original ESI was developed using EFA [12, 76], we decided to first complete two principal axis factor analyses extracting and varimax rotating five factors in an effort to see if the factors were replicable with the ESI-R. In the first analysis, data from all participants were used (N = 4004). In the second, all data except those from the Canadian sample were used (n = 3072). This latter analysis was done in response to the fact that the Canadian sample was employed by MacDonald [12, 76] to develop the ESI. We reasoned that the analysis would be a better indicator of the replicability of the factors. Rotated factor loadings for the two analyses can be seen in Table 9.

**Table 9:** Varimax Rotated Factor Loadings from Principal Axis Factor Analyses of ESI-R Items Using Total Combined Sample and Combined Sample Minus Canadian Sample.

	Total Combined (N = 4004)					Combined minus Canada (n = 3072)				
	1	2	3	4	5	1	2	3	4	5
COS										
Item 1	**.52**	.20	.01	.02	**.54**	**.42**	.21	**.62**	.00	.02
Item 6	**.54**	.17	.00	.12	**.54**	**.48**	.16	**.61**	.01	.15
Item 11	**.53**	.26	.05	.08	**.45**	**.48**	.26	**.50**	.04	.09
Item 16	**.45**	.26	.01	.07	**.38**	**.42**	.28	**.40**	.01	.11
Item 21	**.62**	.25	.06	.11	**.43**	**.59**	.28	**.49**	.06	.10
Item 26	**.55**	.19	−.02	.14	**.52**	**.50**	.19	**.57**	−.01	.16
EPD										
Item 2	.15	**.54**	−.00	.09	.20	.12	**.50**	.22	−.01	.12
Item 7	.07	**.60**	−.07	.18	−.01	.09	**.60**	.01	−.07	.24
Item 12	.11	**.58**	−.01	.27	.06	.12	**.58**	.09	−.00	.26
Item 17	.29	**.65**	.01	.12	.08	**.30**	**.65**	.12	.01	.15
Item 22	.25	**.59**	.00	.10	.11	.25	**.57**	.15	−.01	.11
Item 27	.17	**.64**	−.04	.20	.07	.19	**.64**	.09	−.04	.20
EWB										
Item 3	−.04	−.01	**.57**	−.03	.10	−.07	−.02	.11	**.56**	−.02
Item 8	.15	.03	**.62**	−.01	−.07	.19	.02	−.09	**.62**	−.02
Item 13	−.10	−.04	**.50**	−.05	−.07	−.10	−.00	−.09	**.46**	−.13
Item 18	.05	−.03	**.69**	−.07	.02	.05	−.04	.04	**.70**	−.04
Item 23	.05	−.03	**.56**	−.02	−.06	.08	−.04	−.08	**.54**	−.01
Item 28	.06	.01	**.59**	−.03	.10	.03	.01	.12	**.58**	−.03
PAR										
Item 4	.12	.06	−.06	**.61**	.04	.16	.05	.08	−.07	**.54**
Item 9	−.07	.10	−.05	**.51**	.00	−.07	.11	−.01	−.06	**.45**
Item 14	.05	.17	−.03	**.56**	−.06	.08	.19	−.04	−.03	**.51**
Item 19	.05	.03	−.02	**.42**	.13	.02	.04	.16	−.01	**.32**
Item 24	−.05	.18	−.03	**.51**	.00	−.04	.20	.00	−.03	**.48**
Item 29	.15	.20	−.03	**.56**	.04	.18	.20	.07	−.05	**.52**
REL										
Item 5	**.68**	.05	−.01	−.08	.15	**.67**	.06	.25	−.00	−.03
Item 10	**.66**	.28	.05	.10	.07	**.60**	**.33**	.13	.05	.11
Item 15	**.76**	.11	.05	.01	.15	**.72**	.13	.24	.06	.05
Item 20	**.73**	.19	.05	−.02	.05	**.70**	.22	.12	.05	−.02
Item 25	**.71**	.15	.05	.07	.04	**.69**	.18	.11	.06	.08
Item 30	**.79**	.06	.00	.12	−.04	**.80**	.09	.02	.01	.12

*Note*. COS = Cognitive Orientation toward Spirituality, EPD = Experiential/Phenomenological Dimension, EWB = Existential Well-Being, PAR = Paranormal Beliefs, REL = Religiousness Loadings .30 or higher are in bold font.

For both analyses, elevated factor loadings (i.e., loadings .30 or higher) were found for all ESI-R items in a manner wholly consistent with what MacDonald [12] reported. In particular, items comprising each of the ESI-R dimensions loaded strongly on separate factors with the exception of COS and REL which produced notable loadings on the first factor followed by COS generating strong loadings on a second separate factor. While not reported in this article for the sake of brevity, we also ran the analyses with oblique (oblimin) rotation. Pattern matrices, which provide information on the unique association of a variable to a factor, showed elevated loadings for all items on completely separate factors (i.e., COS and REL did not produce loadings .30 on the same factor).

To determine whether or not the ESI-R dimensions remained replicable across sexes, we completed principal axis factor analyses for all males (n = 1436) and females (n = 2558) separately. The analyses were set to extract five factors and obliquely (oblimin) rotated factor loadings examined. For both sexes, the items comprising the five ESI-R dimensions loaded strongly on separate factors. We did a similar pair of principal axis factors with age. Using a median split, we created a young (i.e., 21 years and younger) group (n = 1995) and an old group (i.e., 22 years and up) (n = 1943). The pattern matrix from both solutions showed loadings consistent with the ESI-R dimensional structure.

For the sake of thoroughness, the factorial stability of the ESI-R dimensions as a function of perceived face validity was also examined. More specifically, responses to ESI item 31 were used to create two groups. One group consisted of all participants who responded strongly disagree, disagree, and neutral to the item (n = 1184) and a second group was comprised of participants who responded agree or strongly agree (n = 2817). Principal axis factor analyses involving the extraction and oblique (oblimin) rotation of five factors produced solutions supportive of the five ESI-R dimensions; pattern matrices showed that all items loaded on clearly identifiable factors.

### Confirmatory Factor Analyses (CFAs)

In order to evaluate the goodness-of-fit of the dimensional model underlying the ESI-R, a series of maximum-likelihood confirmatory factor analyses using Analysis of Moment Structures (AMOS) software were completed. Due to the fact that COS and REL are highly inter-correlated, it was considered worthwhile to also examine the goodness-of-fit of a four factor model wherein these two dimensions were combined into one to see which model (four versus five factors) resulted in better fit.

In total, 18 CFAs were done (9 four factor and 9 five factor), using data for the combined samples first, followed by separate analyses for each country sample. The standardized regression weights along with a variety of fit statistics for the combined sample analysis can be found in Table 10. The overall model fit statistics for the analyses for each country separately can be found in Tables 11 through 14.

**Table 10:** Standardized Regression Weights and Goodness-of-Fit Statistics for Both Four and Five Factor Models of the ESI-R Using the Total Combined Sample (N = 4004).

	Four Factor				Five Factor				
	1	2	3	4	1	2	3	4	5
COS									
Item 1	.72	---	---	---	.76	---	---	---	---
Item 6	.74	---	---	---	.78	---	---	---	---
Item 11	.72	---	---	---	.74	---	---	---	---
Item 16	.62	---	---	---	.65	---	---	---	---
Item 21	.80	---	---	---	.82	---	---	---	---
Item 26	.75	---	---	---	.79	---	---	---	---
EPD									
Item 2	---	.59	---	---	---	.59	---	---	---
Item 7	---	.59	---	---	---	.59	---	---	---
Item 12	---	.64	---	---	---	.64	---	---	---
Item 17	---	.73	---	---	---	.72	---	---	---
Item 22	---	.65	---	---	---	.65	---	---	---
Item 27	---	.70	---	---	---	.70	---	---	---
EWB									
Item 3	---	---	.57	---	---	---	.57	---	---
Item 8	---	---	.62	---	---	---	.62	---	---
Item 13	---	---	.48	---	---	---	.48	---	---
Item 18	---	---	.70	---	---	---	.71	---	---
Item 23	---	---	.57	---	---	---	.57	---	---
Item 28	---	---	.59	---	---	---	.59	---	---
PAR									
Item 4	---	---	---	.61	---	---	---	.61	---
Item 9	---	---	---	.49	---	---	---	.49	---
Item 14	---	---	---	.58	---	---	---	.58	---
Item 19	---	---	---	.41	---	---	---	.41	---
Item 24	---	---	---	.54	---	---	---	.54	---
Item 29	---	---	---	.63	---	---	---	.63	---
REL									
Item 5	.65	---	---	---	---	---	---	---	.69
Item 10	.69	---	---	---	---	---	---	---	.72
Item 15	.74	---	---	---	---	---	---	---	.79
Item 20	.70	---	---	---	---	---	---	---	.76
Item 25	.68	---	---	---	---	---	---	---	.73
Item 30	.68	---	---	---	---	---	---	---	.74

*Note*. Overall fit statistics for the models were as follows: Four factor model: χ^2^ = 5804.96, df = 399, p<,001; χ^2^/df = 14.55; Goodness of Fit Index (GFI) = .89; Adjusted Goodness of Fit Index (AGFI) = .87; Normed Fit Index (NFI) = .87; Relative Fit Index (RFI) = .86; Incremental Fit Index (IFI) = .88; Tucker Lewis Index (TLI) = .87; Comparative Fit Index (CFI) = .88; Root Mean Square Error of Approximation (RMSEA) = .06, p<.001; Standardized Root Mean Residual (SRMR) = .0528. Five factor model: χ^2^ = 3830.64, df = 395, p<.001; χ^2^/df = 9.70; GFI = .94; AGFI = .92; NFI = .92; RFI = .91; IFI = .92; TLI = .92; CFI = .92; RMSEA = .05, p>.05; SRMR = .0477. Δdf from 4 to 5 factor model = 4, Δ χ^2^ from 4 to 5 factor = 1974.32, p<.001. All regression weights significant at p<.05 or lower.

**Table 11:** Goodness of Fit Statistics for Confirmatory Factor Analysis Results for Fully Correlated Four and Five Factor Models for the ESI-R for American and Canadian Samples.

	American		Canadian	
	Four Factor	Five Factor	Four Factor	Five Factor
χ^2^	1065.55***	971.31***	1731.19***	1185.73***
df	399	395	399	395
χ^2^/df	2.67	2.46	4.34	3.00
GFI	.89	.90	.87	.92
AGFI	.87	.88	.85	.91
NFI	.89	.90	.86	.90
RFI	.88	.89	.84	.89
IFI	.93	.94	.89	.93
TLI	.92	.93	.87	.92
CFI	.93	.94	.88	.93
RMSEA	.05	.05	.06***	.05
SRMR	.0583	.0572	.0603	.0521

Note.

***p<.001.

GFI = Goodness of Fit Index; AGFI = Adjusted Goodness of Fit Index; NFI = Normed Fit Index; RFI = Relative Fit Index; IFI = Incremental Fit Index; TLI = Tucker Lewis Index; CFI = Comparative Fit Index; RMSEA = Root Mean Square Error of Approximation; SRMR = Standardized Root Mean Residual. For Americans, Δdf from 4 to 5 factor = 4; Δχ^2^ from 4 to 5 factor = 94.24, p<.001. For Canadians, Δdf from 4 to 5 factor = 4; Δχ^2^ from 4 to 5 factor = 545.46, p<.001. Though not reported, all regression weights for all models significant at p<.05 or lower.

**Table 12:** Goodness of Fit Statistics for Confirmatory Factor Analysis Results for Fully Correlated Four and Five Factor Models for the ESI-R for Indian and Japanese Samples.

	Indian		Japanese	
	Four Factor	Five Factor	Four Factor	Five Factor
χ^2^	990.34***	832.41***	818.87***	705.89***
df	399	395	399	395
χ ^2^/df	2.48	2.11	2.05	1.79
GFI	.91	.93	.76	.80
AGFI	.90	.91	.71	.76
NFI	.82	.85	.67	.72
RFI	.80	.83	.64	.69
IFI	.88	.91	.80	.85
TLI	.87	.90	.78	.83
CFI	.88	.91	.80	.85
RMSEA	.05	.04	.08***	.07***
SRMR	.0480	.0452	.0809	.0737

Note.

***p<.001.

GFI = Goodness of Fit Index; AGFI = Adjusted Goodness of Fit Index; NFI = Normed Fit Index; RFI = Relative Fit Index; IFI = Incremental Fit Index; TLI = Tucker Lewis Index; CFI = Comparative Fit Index; RMSEA = Root Mean Square Error of Approximation; SRMR = Standardized Root Mean Residual. For Indians, Δdf from 4 to 5 factor = 4; Δχ^2^ from 4 to 5 factor = 157.93, p<.001. For Japanese, Δdf from 4 to 5 factor = 4; Δχ^2^ from 4 to 5 factor = 112.98, p<.001. Though not reported, all regression weights for all models significant at p<.05 or lower.

**Table 13:** Goodness of Fit Statistics for Confirmatory Factor Analysis Results for Fully Correlated Four and Five Factor Models for the ESI-R for Korean and Polish Samples.

	Korean		Polish	
	Four Factor	Five Factor	Four Factor	Five Factor
χ^2^	1842.66***	1500.52***	1044.71***	975.74***
df	399	395	399	395
χ^2^/df	4.62	3.80	2.62	2.47
GFI	.79	.84	.84	.85
AGFI	.76	.81	.82	.82
NFI	.81	.84	.77	.78
RFI	.79	.83	.75	.76
IFI	.84	.88	.84	.86
TLI	.83	.87	.83	.84
CFI	.84	.88	.84	.86
RMSEA	.08***	.07***	.06***	.06***
SRMR	.0723	.0661	.0887	.0882

Note.

***p<.001.

GFI = Goodness of Fit Index; AGFI = Adjusted Goodness of Fit Index; NFI = Normed Fit Index; RFI = Relative Fit Index; IFI = Incremental Fit Index; TLI = Tucker Lewis Index; CFI = Comparative Fit Index; RMSEA = Root Mean Square Error of Approximation; SRMR = Standardized Root Mean Residual. For Koreans, Δdf from 4 to 5 factor = 4; Δχ^2^ from 4 to 5 factor = 342.14, p<.001. For Polish, Δdf from 4 to 5 factor = 4; Δχ^2^ from 4 to 5 factor = 68.97, p<.001. Though not reported, all regression weights for all models significant at p<.05 or lower.

**Table 14:** Goodness of Fit Statistics for Confirmatory Factor Analysis Results for Fully Correlated Four and Five Factor Models for the ESI-R for Slovakian and Ugandan Samples.

	Slovakian		Ugandan	
	Four Factor	Five Factor	Four Factor	Five Factor
χ^2^	804.35***	719.59***	775.62***	729.05***
df	399	395	399	395
χ^2^/df	2.02	1.82	1.94	1.85
GFI	.76	.79	.90	.90
AGFI	.72	.76	.88	.89
NFI	.63	.67	.76	.78
RFI	.59	.63	.74	.75
IFI	.77	.82	.87	.88
TLI	.74	.79	.85	.87
CFI	.76	.81	.87	.88
RMSEA	.08***	.07***	.05	.04
SRMR	.0869	.0852	.0582	.0573

Note.

***p<.001.

GFI = Goodness of Fit Index; AGFI = Adjusted Goodness of Fit Index; NFI = Normed Fit Index; RFI = Relative Fit Index; IFI = Incremental Fit Index; TLI = Tucker Lewis Index; CFI = Comparative Fit Index; RMSEA = Root Mean Square Error of Approximation; SRMR = Standardized Root Mean Residual. For Slovakians, Δdf from 4 to 5 factor = 4; Δχ^2^ from 4 to 5 factor = 84.76, p<.001. For Ugandans, Δdf from 4 to 5 factor = 4; Δχ^2^ from 4 to 5 factor = 46.57, p<.001. Though not reported, all regression weights for all models significant at p<.05 or lower.

In all analyses for both four and five factor models, inspection of parameter estimates indicated that all regression weights (i.e., factor loadings) came out statistically significant as did all error variances (p<.05 or lower). Alternatively, examination of covariances (i.e., the intercorrelations between ESI-R dimensions), revealed some differences across models and samples. For instance, all covariances emerged significant with the total combined sample in both four and five factor models. However for the Canadian sample, the four-factor model produced nonsignificant estimates for all covariances involving EWB and in the five-factor model, five covariances were nonsignificant (i.e., all involving EWB, and PAR-REL). For the American sample, two covariances were nonsignificant in the four-factor model (i.e., EPD-EWB, and combined COS/REL-PAR) and three in the five-factor model (i.e., PAR-REL, COS-PAR, and EPD-EWB). For the four-factor model for the Polish sample, one covariance estimate was nonsignificant (i.e., between combined COS/REL-EWB) and two were nonsignificant in the five-factor model (i.e., EWB-REL, and COS-EWB). In the Slovakian sample, four emerged nonsignificant in the four-factor model (i.e., all with PAR and combined COS/REL-EWB), and six in the five-factor model (i.e., all with PAR, EWB-REL, and COS-EWB). In the Ugandan sample, two covariance estimates were not significant (i.e., EPD-EWB and combined COS/REL-PAR) and three in the five-factor model (i.e., PAR-REL, COS-PAR, and EPD-EWB). In the Indian sample, the covariance estimate for the combined COS/REL and EWB was not significant in the four-factor model while in the five-factor model, two came out non-significant (i.e., EWB-REL and COS-EWB). In the Korean sample, two covariances were found to be nonsignificant (i.e., EPD-EWB and combined COS/REL-EWB), and three for the five-factor model (i.e., EWB-REL, EPD-EWB, and COS-EWB). Lastly, for the Japanese sample, two covariance estimates were not significant in the four-factor model (i.e., EPD-EWB, and combined COS/REL-EWB) and three in the five factor model (i.e., EWB-REL, EPD-EWB, and COS-EWB).

When comparing the overall model fit statistics between four and five factor models for the total combined sample and for each country sample, the correlated five factor model emerged superior as reflected in significant reduction in chi-square values (i.e., the five factor model produced significantly lower chi-square values than the four factor model). Based on this, the five factor model became the focus of our remaining analyses.

Notwithstanding the findings supporting the five-factor model, closer examination of the fit indices suggests that overall model fit was not wholly satisfactory across all analyses. On the positive side, fit statistics for the combined total sample, Canadians, Americans, and Indians provide reasonably good support for model fit (e.g., despite chi-square being significant and the chi-square/df ratio exceeding 3.0, GFI, AGFI, NFI, RFI, IFI, TLI, and CFI are close to or exceed .90; RMSEA is lower than .08 and SRMR is close to or lower than .05, [42, 44, 96]). The fit statistics for the Ugandan sample, while not as compelling, also appear to be at least somewhat adequate. On the other hand, for the remaining samples, all of which used translated versions of the ESI, fit statistics are less consistently supportive of good fit of the five factor model.

To identify possible causes for the poorer model fit (i.e., model misspecification), modification indices were examined for the Polish, Slovakian, Korean, and Japanese samples. While a number of modification indices were generated, none of them indicated that any part of the model for these four samples could be respecified in a manner that made rational sense. For instance, there was nothing pointing to correlated error variances suggesting possible systematic measurement error due to unintended overlap in item content [45]. Similarly, there were no modification indices which strongly supported the re-assignment of an ESI-R item from one dimension to another in a way that would be defendable from a conceptual point of view or would generalize beyond a single country sample.

Finally, to determine the extent to which parameter estimates for the five-factor model are stable and generalizable beyond the current samples, maximum likelihood bootstrap analyses were completed for the total combined sample and each country sample. Examination of 90% bias corrected confidence intervals for estimates generated from 1000 bootstrap samples revealed non-zero value ranges (i.e., the confidence interval did not contain zero; Byrne [42, 96] notes that if zero is contained in the interval then one cannot reject the hypothesis that the parameter value for the population is zero) for all variances and regression weights for all samples. Confidence intervals for nonsignificant covariances as reported above, conversely, were found to include zero.

### Test of Measurement Invariance

As the most rigorous test of the ESI-R, we completed a series of CFA analyses wherein a freely estimated five factor model was compared to a model with parameter estimates constrained to equality and the change in goodness of fit evaluated simultaneously across each country sample. Based upon the previous CFAs done for each country separately, it was decided that we would test a model with only the factor loadings constrained as factor inter-correlations varied across samples and appeared likely to contribute to poor model fit. Table 15 presents the freely estimated standardized regression weights for the country samples along with essential fit statistics. Table 16 provides an overview of the model invariance testing analyses that were done.

**Table 15:** Standardized Regression Weights for All Country Samples from Simultaneous Multiple Group CFA of Five Factor ESI-R Model with all Parameters Freely Estimated.

	A	C	I	J	K	P	S	U
COS								
Item 1	.90	.83	.72	.56	.69	.73	.77	.64
Item 6	.79	.69	.72	.53	.80	.69	.76	.75
Item 11	.84	.75	.54	.52	.85	.72	.61	.57
Item 16	.63	.60	.52	.63	.76	.66	.60	.56
Item 21	.90	.78	.75	.73	.86	.70	.85	.81
Item 26	.77	.74	.74	.58	.80	.69	.68	.75
EPD								
Item 2	.74	.69	.49	.64	.68	.47	.66	.40
Item 7	.56	.50	.45	.74	.75	.73	.39	.44
Item 12	.69	.65	.50	.85	.73	.65	.36	.48
Item 17	.74	.67	.61	.83	.81	.72	.73	.70
Item 22	.70	.65	.61	.73	.82	.50	.56	.54
Item 27	.72	.70	.57	.78	.78	.70	.65	.60
EWB								
Item 3	.67	.64	.50	.58	.68	.56	.49	.43
Item 8	.63	.61	.62	.56	.64	.78	.61	.55
Item 13	.68	.65	.41	.54	.63	.29	.30	.48
Item 18	.74	.69	.66	.67	.70	.81	.64	.54
Item 23	.71	.63	.51	.49	.37	.63	.58	.48
Item 28	.66	.62	.60	.61	.63	.60	.57	.52
PAR								
Item 4	.65	.75	.49	.68	.55	.36	.37	.46
Item 9	.57	.57	.41	.57	.50	.63	.51	.49
Item 14	.61	.64	.43	.46	.66	.66	.60	.20
Item 19	.55	.64	.37	.68	.32	.24	.41	.43
Item 24	.61	.64	.46	.66	.65	.53	.61	.35
Item 29	.62	.73	.49	.70	.69	.48	.45	.19
REL								
Item 5	.65	.62	.54	.41	.70	.75	.76	.61
Item 10	.83	.83	.63	.74	.84	.37	.71	.61
Item 15	.84	.80	.66	.74	.75	.74	.71	.60
Item 20	.73	.79	.63	.77	.81	.71	.68	.59
Item 25	.78	.76	.57	.82	.66	.70	.57	.38
Item 30	.70	.73	.66	.38	.80	.67	.76	.47

*Note*. All regression weights significant at p<.05 or lower. For groups, A = American, C = Canadian, I = Indian, J = Japanese, K = Korean, P = Polish, S = Slovakian, U = Ugandan. For ESI Dimensions, COS = Cognitive Orientation toward Spirituality, EPD = Experiential/ Phenomenological Dimension, EWB = Existential Well-Being, PAR = Paranormal Beliefs, REL = Religiousness. Model Fit Statistics: χ^2^ = 7623.81, df = 3160, χ ^2^/df = 2.41, CFI = .90, RMSEA = .019, p>.05.

**Table 16:** Tests of Measurement Invariance for the ESI-R.

Model	Groups	Comparison Model	CFI	RMSEA	χ ^2^	df	Δ χ ^2^	Δ df
1. Baseline five factor	All	---	.90	.019	7623.81	3160	---	---
2. Loadings constrained equal	All	Model 1	.88	.020	8616.46	3335	992.65	175
3. Baseline five factor	C, A, I, U	---	.92	.022	3718.68	1580	---	---
4. Loadings constrained equal	C, A, I, U	Model 3	.92	.023	3998.72	1655	280.04	75
5. Baseline five factor	C, A, I	---	.93	.026	2989.52	1185	---	---
6. Loadings constrained equal	C, A, I	Model 5	.92	.027	3180.18	1235	190.66	50
7. Baseline five factor	C, A	---	.93	.034	2157.12	790	---	---
8. Loadings constrained equal	C, A	Model 7	.93	.034	2223.94	815	66.82	25
9. Baseline five factor	C, I	---	.93	.031	2018.21	790	---	---
10. Loadings constrained equal	C, I	Model 9	.92	.031	2104.59	815	86.38	25
11. Baseline five factor	A, I	---	.93	.031	1803.77	790	---	---
12. Loadings constrained equal	A, I	Model 11	.92	.033	1948.04	815	144.27	25
13. Baseline five factor	P, S	---	.84	.045	1695.92	790	---	---
14. Loadings constrained equal	P, S	Model 13	.83	.046	1788.86	815	92.94	25
15. Baseline five factor	J, K	---	.87	.049	2207.03	790	---	---
16. Loadings constrained equal	J, K	Model 15	.86	.051	2362.09	815	156.06	25

*Note*. CFI = Comparative Fit Index, RMSEA = Root Mean Square Error of Approximation. For groups, A = American, C = Canadian, I = Indian, J = Japan, K = Korea, P = Polish, S = Slovakian, U = Ugandan. All chi-square values significant at p<.001.

For the first analysis, the baseline model (i.e., the model with freely estimated loadings) was compared to the constrained model for all eight countries. For the baseline model, CFI and RMSEA values reflect adequate fit though the chi-square emerged significant. For the constrained model, chi-square remained significant and the CFI value falls below .90. Comparison of the change in chi-square across the two models indicates that the constrained model reflects a significantly poorer fit suggesting non-invariance.

While Byrne [42] recommends systematically modifying and testing the constraints in a model to identify elements that are invariant versus non-invariant across samples, we reasoned that such an approach was not practical in the case of our study as there are simply too many comparisons to be made with a 30 item test across eight samples. Instead, we adopted the approach of examining the same constrained model with different sets of country samples as we saw this as being more consistent with our hypotheses. In this vein, we evaluated our baseline model to a constrained model using the four samples which completed the ESI-R in English (i.e., American, Canadian, Indian, and Ugandan). The CFI and RMSEA for both models reflect adequate fit though the change in chi-square still emerged significant. We next used just the American, Canadian, and Indian samples with the same result (i.e., good CFA and RMSEA but significant change in chi-square). We did the same analyses comparing just Americans and Canadians, Americans and Indians, and Canadians and Indians, respectively. In all cases, the same pattern of findings were obtained; the baseline model and constrained model showed adequate CFI and RMSEA values but the change of chi-square came out significant with the constrained model always demonstrating poorer fit. We then completed analyses comparing the Polish and Slovakian samples, and the Korean and Japanese samples, respectively. With these, though the RMSEA was still satisfactory, the CFI was below .90 for both the baseline and constrained models. Regardless, in both instances, the constrained model was found to produce a significantly poorer fit.

### Analyses Involving the Spirituality Adjective List (SAL) and ESI-R

To further evaluate whether or not the findings reported thus far were the product of the type of test used (and by association the type of item and test development strategy), exploratory principal component analyses were used to examine the internal structure of the SAL with the American, Indian, and Ugandan samples. In all cases, the analyses were set to extract and orthogonally (varimax) rotate five components. To ascertain their association to the ESI dimensions, regression based component scores were calculated and used in a correlational analysis. The rotated component loading coefficients can be found in Table 17. The correlations of the component scores to the ESI-R dimensions are reported in Table 18.

**Table 17:** Varimax Rotated Component Loadings for the Spirituality Adjective List Items Across American, Indian, and Ugandan Samples.

	American (n = 257)					Indian (N = 718)					Ugandan (N = 447)				
SAL	1	2	3	4	5	1	2	3	4	5	1	2	3	4	5
Item 1	**.44**	.04	**.41**	−.02	.04	**.44**	.22	**.35**	−.29	.00	**.48**	.20	.11	.17	.29
Item 2	**.45**	.10	**.37**	.13	.15	**.30**	.20	**.54**	−.11	−.12	**.46**	**.35**	.01	.20	.06
Item 3	**.62**	−.03	−.00	.21	.12	.09	**.57**	.09	.11	−.00	.09	**.30**	**.46**	−.07	.00
Item 4	**.52**	.13	.03	.27	.02	.07	**.38**	.10	**.46**	.08	.07	**.42**	**.35**	.06	.15
Item 5	**.72**	−.00	.23	−.03	−.08	−.00	**.52**	.06	**.31**	−.03	−.07	**.62**	.12	.22	−.10
Item 6	.13	.28	**.59**	.10	.14	.25	.09	.17	.24	**.35**	**.32**	**.36**	−.02	.07	.11
Item 7	.08	**.58**	**.32**	.14	.03	**.34**	.24	.01	.27	.21	.28	**.43**	**.34**	**−.30**	−.05
Item 8	.09	**.78**	.24	−.06	.11	**.69**	.08	−.01	.07	.10	**.58**	.10	.19	−.12	−.21
Item 9	.07	**.78**	.23	.02	.19	**.78**	−.02	.08	.09	.11	**.76**	.07	.16	−.03	−.08
Item 10	.06	**.78**	−.00	.07	.26	**.75**	−.03	.14	.02	.19	**.68**	.04	.06	.14	.14
Item 11	**.81**	.08	.25	−.01	−.03	.07	**.69**	.02	.10	.01	.05	**.58**	.10	.10	−.09
Item 12	**.80**	.07	.07	.06	−.02	.09	**.69**	.02	−.02	−.07	.11	**.56**	.06	.13	−.16
Item 13	.00	.00	.10	.06	**.74**	.17	−.13	−.18	−.05	**.57**	−.14	−.03	.03	−.12	**.71**
Item 14	−.14	.20	**.33**	**.34**	.24	−.00	−.03	**.62**	.09	.20	.27	−.05	−.06	**.53**	.15
Item 15	**.64**	.18	.21	.28	−.02	−.08	**.57**	.20	.17	.14	.09	.26	**.43**	.23	.19
Item 16	−.00	**.31**	.02	−.01	**.73**	.25	.07	.08	−.19	**.65**	**.51**	.11	.04	.12	**.47**
Item 17	**.70**	.16	**.35**	.05	.01	.06	**.44**	.22	.10	.28	.27	**.54**	.14	.11	.23
Item 18	**.42**	.29	**.55**	.06	.07	.07	.13	.22	.12	**.44**	.23	**.49**	.10	.06	.26
Item 19	.14	−.10	**.44**	**.35**	.05	.12	.17	.29	.24	−.17	.05	.21	.08	**.56**	.02
Item 20	.09	.04	.28	**.72**	−.05	.05	.16	.16	**.55**	−.00	.01	.15	**.41**	**.55**	−.06
Item 21	.16	.18	**.57**	.14	−.04	−.01	−.08	**.44**	.08	.20	.14	.14	.04	**.55**	.01
Item 22	.15	**.54**	**.50**	−.09	.20	**.51**	−.03	.16	.17	**.35**	**.53**	.15	.16	.09	.16
Item 23	.13	**.71**	.09	−.10	.08	**.48**	−.01	.01	.02	.15	**.56**	−.02	.10	.08	−.01
Item 24	−.01	.20	.24	.01	**.76**	**.46**	.06	−.03	−.02	**.48**	.11	−.08	−.03	.10	**.67**
Item 25	**.52**	.22	**.36**	.07	.10	−.00	.16	**.58**	.13	.13	.25	**.42**	.03	**.49**	.23
Item 26	**.55**	.11	.21	.18	−.01	−.00	**.36**	**.40**	−.05	.07	.05	.05	.23	**.31**	**.33**
Item 27	.03	**.42**	−.02	.03	**.62**	.26	.13	.10	−.15	**.67**	**.42**	.23	−.02	.12	**.45**
Item 28	.05	.08	**.34**	.06	.21	.05	.00	.17	.16	−.05	.08	.01	**.30**	−.08	.02
Item 29	.18	−.13	**.44**	**.46**	.07	.02	.08	**.43**	**.40**	−.07	.03	.07	**.37**	.23	−.00
Item 30	**.43**	**.38**	**.58**	−.02	.06	.26	.13	.14	**.37**	**.44**	**.35**	**.56**	.22	.01	.00
Item 31	**.36**	**.59**	**.44**	.03	.14	**.39**	.27	**.36**	.20	.02	**.57**	**.42**	.15	.02	.11
Item 32	**.52**	.14	**.43**	.16	.06	.27	.18	**.52**	.01	−.03	**.35**	.24	.20	**.34**	.10
Item 33	**.30**	.15	.06	**.80**	.04	.18	.08	**.41**	**.40**	−.13	.11	.11	**.57**	**.48**	−.13
Item 34	.22	.11	.03	**.78**	−.02	.10	**.34**	.06	**.55**	.00	.10	.05	**.69**	.19	−.04
Item 35	**.39**	−.03	.06	**.57**	.13	.03	**.31**	−.01	**.67**	.00	.10	**.30**	**.55**	.26	.05
Item 36	**.39**	.02	**.59**	.18	−.07	−.00	.13	**.48**	**.38**	.17	.05	**.51**	.27	.24	.02
Item 37	.04	**.84**	−.07	.16	.16	**.82**	.01	.05	.07	.12	**.59**	.08	.07	.16	−.00
Item 38	**.67**	.11	.01	.28	−.09	.07	**.61**	.01	.25	.08	.15	.13	**.76**	−.03	.03
Item 39	**.75**	.09	−.00	.08	.00	.03	**.57**	.10	.17	.06	.20	**.49**	**.44**	−.03	.08
Item 40	.22	**.77**	.04	.18	−.00	**.48**	.27	.12	.10	.09	**.47**	**.32**	.02	.18	−.03

*Note*. Items with loadings .30 or higher in bold.

**Table 18:** Product-Moment Correlations Between Regression-Based SAL Component Scores and ESI-R Dimensions.

	ESI Dimensions				
Components	COS	EPD	EWB	PAR	REL
American					
Comp 1	.16*	.02	.65***	−.09	.14*
Comp 2	.67***	.25***	.06	−.09	.85***
Comp 3	.44***	.48***	.22***	.14*	.30***
Comp 4	.01	.09	−.05	.05	.00
Comp 5	.16*	.28***	−.18**	.60***	.11
Indian					
Comp 1	.56***	.26***	.02	.13**	.77***
Comp 2	−.00	.01	.61***	−.02	.05
Comp 3	.20***	.17***	.03	.01	.12**
Comp 4	.10*	.10**	−.06	−.04	.03
Comp 5	.39***	.41***	−.06	.55***	.29***
Ugandan					
Comp 1	.71***	.30***	.13**	−.13**	.72***
Comp 2	.21***	.17***	.27***	.00	.20***
Comp 3	.08	.05	.10*	−.10*	.05
Comp 4	.11*	.01	.10*	.06	−.05
Comp 5	.12*	.18***	.08	.39***	.05

*Note*. Comp = Component. For ESI, COS = Cognitive Orientation toward Spirituality, EPD = Experiential/Phenomenological Dimension, EWB = Existential Well-Being, PAR = Paranormal Beliefs, REL = Religiousness.

*p<.05

**p<.01

***p<.001.

Examination of Table 17 reveals that for all three country samples, all five components house elevated loadings (i.e., loadings .30 or greater) for at least four SAL items. Also, while there appear to be a large number of differences in the pattern of item loadings across the three countries, there are also some points of similarity which find corroboration in the correlations with the ESI-R as per Table 18. In particular, component one for the American sample and component two for both the Indian and Ugandan samples produce their highest correlation with ESI-R Existential Well-Being (r = .65, .61, and .27, respectively, all p<.001) and have common loadings from items 3, 4, 5, 11, 12, 17, 35, and 39. All of these items concern positive self-evaluation (e.g., understanding self, feeling happy and content, sense of completeness or wholeness, identification of self as moral). Component 2 from the American sample and component one from both the Indian and Ugandan samples produce their highest correlation with ESI-R Religiousness (r = .85, .77, and .72, all p<.001) followed by ESI-R Cognitive Orientation toward Spirituality (r = .67, .56, and .71, all p<.001) and share elevated loadings with items 8, 9, 10, 22, 23, 31, 37, and 40. The content of these items revolve around putative religious beliefs and behavior (e.g., self identification as being religious and blessed, participation in activities such as prayer and church services, and beliefs in God or a higher power). Component five from all three analyses show their strongest correlation with ESI-R Paranormal Beliefs (r = .60, .55, and .39, p <.001 for American, Indian, and Ugandan samples, respectively) and have strong loadings from items 13, 16, 24, and 27. All four of these items have content which has obvious ties to paranormal beliefs (e.g., belief in life after death, supernatural powers, and ghosts). Components 3 and 4 from all three solutions show much less similarity to each other in terms of the pattern of high item loadings and their correlations with the ESI-R dimensions. In terms of the correlations, for the Indian and Ugandan samples, though some statistically significant coefficients were obtained, the correlations are generally of small magnitude. For the American sample, no significant correlations were obtained between component four and any ESI-R dimension but, unlike the other samples, the correlations with component three were statistically significant and of medium magnitude between four of the five ESI-R dimensions (i.e., all but ESI Paranormal Beliefs). Taken as a whole, these findings suggest that three of the ESI-R dimensions (i.e., Religiousness, Existential Well-Being, and Paranormal Beliefs) find fairly good representation in the SAL components. ESI-R Cognitive Orientation toward Spirituality (COS) also seems to be implicated as it is highly correlated with the SAL component reflective of Religiousness in a manner similar to what is seen in the ESI-R. However, unlike the ESI-R where COS is a discrete dimension, this is not observed with the SAL. Only the ESI-R Experiential-Phenomenological Dimension does not find any approximate manifestation in the SAL items.

In consideration of these results, we elected to create three subscales with the SAL items common to all three country samples so as to see if they function adequately in terms of reliability and if they produce a similar array of associations as found with the ESI-R. Descriptive and reliability statistics, SAL subscale inter-correlations, and correlations with the ESI-R dimensions can be seen in Table 19.

**Table 19:** Descriptive Statistics, Reliabilities, SAL Subscale Inter-correlations and Correlations with ESI-R Dimensions Across American, Indian, and Ugandan Samples.

	SAL Subscales								
	American			Indian			Ugandan		
	REL	EWB	PAR	REL	EWB	PAR	REL	EWB	PAR
Descriptives									
Mean	23.31	24.05	10.76	21.77	21.75	7.05	25.06	23.19	9.98
S.D.	6.37	4.39	3.41	5.34	4.12	3.64	4.20	3.95	3.07
Reliability									
Alpha	.90	.87	.77	.82	.76	.72	.79	.76	.58
Mean CISr	.70	.62	.57	.54	.46	.51	.51	.46	.37
Correlations									
Age	.12	.10	.04	−.11**	−.00	.04	.03	−.04	.12*
Sex	.23***	.08	.14*	.27***	.03	−.01	.23***	.07	.02
SAL-EWB	.30***			.24***			.44***		
SAL-PAR	.47***	.07		.47***	.08*		.31***	.17***	
ESIR-COS	.78***	.27***	.39***	.64***	.12**	.47***	.71***	.33***	.32***
ESIR-EPD	.40***	.15*	.40***	.36***	.12**	.40***	.31***	.19***	.24***
ESIR-EWB	.20**	.64***	−.11	.07	.52***	−.01	.20***	.36***	.13**
ESIR-PAR	.07	−.04	.55***	.21***	.02	.59***	−.11*	−.04	.37***
ESIR-REL	.92***	.25***	.38***	.81***	.16***	.49***	.73***	.29***	.25***

*Note*. Mean CISr = Mean corrected item-to-scale total correlation. For SAL, REL = Religiousness comprised of items 8, 9, 10, 22, 23, 31, 37, and 40; EWB = Existential Well-Being comprised of items 3, 4, 5, 11, 12, 17, 35, and 39; PAR = Paranormal Beliefs comprised of items 13, 16, 24, and 27. ESIR-COS = ESI-R Cognitive Orientation toward Spirituality; ESIR-EPD = ESI-R Experiential/Phenomenological Dimension; ESIR-EWB = ESI-R Existential Well-Being; ESIR-PAR = ESI-R Paranormal Beliefs; ESIR-REL = ESI-R Religiousness. For sex, male coded 0, female coded 1.

*p<.05

**p<.01

***p<.001.

Analyses show a pattern of findings that are generally consistent with what was found for the ESI-R though some deviations are noted. First, one-way ANOVAs uncovered significant findings for all three SAL subscales with effect sizes generally on par with what was seen with ESI-R dimensions assessing the same constructs (SAL Religiousness: F(2, 1419) = 54.84, p<.001, η^2^ = .07; SAL Existential Well-Being: F(2, 1419) = 35.66, p<.001, η^2^ = .05; SAL Paranormal Beliefs: F(2, 1419) = 160.53, p<.001, η^2^ = .19). Post-hoc analyses (Scheffe test) showed that all country samples were significantly different from one another for all three SAL subscales. Second, reliability analyses indicate that the three SAL subscales produce mostly satisfactory inter-item consistency coefficients and good corrected item-to-scale total correlations. The only exception was the SAL Paranormal Beliefs subscale which generated a marginal alpha for the Ugandan sample. Third, in terms of associations with demographic variables, akin to the ESI-R, the SAL subscales produce a pattern of small correlations with age. With sex, SAL Religiousness produced significant and moderately sized correlations in all three country samples while the remaining two SAL subscales generated small coefficients. Fourth, for all three country samples, the SAL subscales produced correlations with the ESI-R dimensions supportive of convergent validity (e.g., scales of the same name from both measures produce their strongest associations with each other), though not discriminant validity (e.g., SAL subscales produce moderate to strong correlations with more than one ESI-R dimension in virtually all cases). The only results which appear to diverge from those with the ESI-R concern the inter-correlations of SAL subscales; for all three country samples, SAL Religiousness was found to produce moderately sized significant correlations with both SAL Existential Well-Being and SAL Paranormal Beliefs. Also, the correlations between SAL Paranormal Beliefs and SAL Existential Well-being, while small in magnitude, are positive while they came out negative with the ESI-R.

## Discussion

This investigation offers a wealth of information that has substantive ramifications for the cross-cultural study of spirituality. Related to our research expectations, results provide generally satisfactory support for the first three hypotheses, no support for our fourth expectation and mixed support for our fifth. To elaborate, consistent with the first hypothesis, the ESI-R appears to demonstrate reasonably good face validity with a notable number of participants responding “agree” or “strongly agree” to ESI-R item 31 (i.e., “this test appears to be measuring spirituality”) and relatively few across the country samples responding “disagree” or “strongly disagree.” Though the interpretation of this outcome is tempered by the significant ANOVA result which found a multitude of pairwise differences across country samples, the overall implication is that the ESI-R is seen by substantial number of people with differing cultural and/or linguistic backgrounds as measuring something akin to what they consider as “spiritual.”

As per the second hypothesis, the ESI-R was found to produce satisfactory reliability coefficients and corrected item-to-scale correlations for the pooled sample and mostly adequate alphas and correlations for the country samples separately. In terms of inter-correlations between dimensions, all samples generated significant correlations of moderate to high magnitude between COS, REL and EPD, and EPD and PAR. A noteworthy and unexpected trend, however, was observed with the samples that had the lowest representations of Christians (i.e., Indian, Japanese, Korean). Specifically, significant associations were found between PAR and REL, and PAR and COS which were not observed with the remaining samples that were more Christian dominant. Lastly, correlations of the ESI-R dimensions with demographic variables show similar trends across most country samples; with the exception of the Koreans, coefficients were of generally low magnitude and in the direction where age and females showed associations with higher scores (especially with COS and REL). In the case of the Korean sample, correlations tended to be of more moderate size with both age and sex.

Third, evidence of factor replicability, configural invariance, and superiority of a five factor over a four factor model was provided by the EFAs and CFAs with the pooled sample and with the CFAs done for each country separately. With the former analyses, ESI-R items loaded in a manner very similar to MacDonald [12] both with and without the Canadian sample included in the analysis. In the CFAs, while loadings were significant for all items in both the four and five factor models, the five factor model displayed a significantly better fit to the data as reflected in both the change in chi-square and virtually all other fit indices. For CFAs involving the country samples, item loadings were ubiquitously significant for all models tested, but the five factor model consistently demonstrated better goodness-of-fit. With that stated, inspection of numerous fit indices for the five factor model for each country separately suggests that the model demonstrates elements of misfit for the Korean, Japanese, Polish, Slovakian, and Ugandan samples. Nevertheless, modification indices were examined and there were no indications of how the model could be meaningfully respecified in a congruent manner across all samples, so the five factor model appears to be the most defendable.

The fourth hypothesis which predicted that the ESI-R would demonstrate measurement invariance was not corroborated by our findings. Tests comparing an equality constrained model to a freely estimated correlated five factor model consistently revealed that the constrained model had significantly poorer fit. Also, significant differences were found at the item and dimension level as a function of country as per ANOVA findings.

The fifth research expectation, namely that the SAL would produce an internal structure which emulates the ESI-R dimensions found some support as factors generally corresponding to ESI-R Religiousness, Paranormal Beliefs, and Existential Well-Being were found. ESI-R Cognitive Orientation toward Spirituality was also observed to generate notable associations with the same factor as Religiousness, a result which seems copasetic with what we found with each and every country sample in this study. Though the ESI-R Experiential/Phenomenological Dimension was not clearly represented in the SAL factor structure, examination of SAL items reveals a complete absence of content related to subjective spiritual experiences, so this finding makes some sense. In addition, when three subscales were created for the SAL, they demonstrated a pattern of findings in terms of reliability, and correlations with demographic variables mostly similar to what was seen for the ESI-R. One point of divergence between the SAL and ESI-R, however, concerned the inter-correlations of subscales. With the SAL, Religiousness was found to produce significant and moderately sized coefficients with both Paranormal Beliefs and Existential Well-Being for all three country samples. Such associations were not found with the ESI-R. All the same, many of the findings with the SAL seem to be fall in line with our expectations.

So what do all of these findings tell us? In general, it appears that when defined and assessed quantitatively, spirituality may be viewed as a viable concept which empirically behaves in a similar manner across cultures. It also seems that spirituality is best treated as a multidimensional construct made up of related but unique components. While the number of these components was found to vary as a function of the inclusiveness item content seen in measures employed in the present study, based upon the results using the Expressions of Spirituality Inventory-Revised, it may be argued that spirituality is comprised of at least five dimensions. At the same time, the results indicate quite clearly that spirituality is not a concept that “transcends” culture and holds a firm universality of meaning. Rather, it seems the opposite holds true; the specific meaning ascribed to spirituality appears to be intrinsically bound by culture and cannot be fully understood without consideration given to cultural factors. That is, while there are similarities, spirituality is not the same across cultures. The significance of our results seem quite apparent—nomothetic approaches to the study of spirituality are at best incomplete and at worst run the risk of misrepresenting the construct and any associations claimed to exist between it and other aspects of functioning. Accordingly, a concrete recommendation for future research is for investigators to be mindful of the role and influence of culture and to augment quantitative methods based solely on self-report questionnaires with other hard quantitative procedures (e.g., direct behavioral observation, neurophysiological measures; see [97] regarding the latter) and, as importantly, qualitative data gathering strategies which permit for culture-specific content to be procured and concurrently analyzed. We offer this suggestion not just for research using samples drawn from different nation states but also for studies using samples of different ethnicities obtained within more pluralistic societies (e.g., United States, Canada, United Kingdom). As well, we strongly suggest that any and all empirical findings generated with samples obtained from one culture be tested and replicated with samples taken from several other cultures prior to making any claims regarding generalizable scientific knowledge. In this vein, we encourage investigators throughout the world to challenge and expand upon our findings using samples from the same and different cultures. The manner in which we report our results in this paper (e.g., item and dimension descriptives, factor loadings for pooled and separate samples) was deliberately done in a way so as to facilitate direct comparisons with other samples.

Our findings hold other important implications. First, while not demonstrating measurement invariance, the five dimensional model of MacDonald [12] did receive support for its configural invariance and its pattern of associations with the SAL were similar across cultures. Given that the dimensions have been found to be differentially related to psychological functioning [91], it seems reasonable to conjecture that such results may also be manifested in studies with different cultures. Though this is an empirical question which would be best answered by future cross-cultural research, when considering the current state of the science, it seems necessary if not prudent at the present time to discourage investigators and practitioners from characterizing the association of spirituality to functioning in solely positive terms [98] as it appears likely that any link found may be a product of how spirituality is defined and measured [99]. Future studies need to either be more inclusive in terms of what they are considering to be spirituality or acknowledge up front that they are only focusing on specific facets of the construct domain.

A second notable implication concerns the results involving the demographic variables. In particular, age and sex were found to be significantly correlated with at least one dimension or scale from both the ESI-R and the SAL for every cultural sample. In addition, significant interaction effects were obtained between culture and age and/or sex for all ESI dimensions save Existential Well-Being. Considering past research which has uncovered such associations with the original ESI and other measures [18, 100, 101], it seems reasonable to argue that spirituality may not only differ in precise meaning across cultures but also across age and sex and as a function of the interaction of all three of these variables (however, see [17]). Clearly, more studies are needed to fully substantiate this interpretative possibility. Nevertheless, it appears as though our findings further challenge assertions regarding the universality of spirituality and call attention to the need to better account for its diversity of experience and expression in research and application as a function of individual differences [102].

There is one other aspect of our results which deserves mention and it concerns Existential Well-Being. Though many operationalizations of spirituality have been criticized on the grounds that they are confounded with well-being [14] and available evidence suggests that ESI-R Existential Well-Being (EWB) may be best treated as something separate from spirituality [15], results in the present study give some reason to reflect more on the issue. In particular, while we observed that ESI-R EWB was modestly associated to the other ESI-R dimensions for all cultures, our findings with the SAL indicate that something akin to existential well-being comprises a replicable component which correlates moderately with other elements of spirituality, most notably Religiousness and Cognitive Orientation toward Spirituality. Since the SAL was developed based upon written narratives describing a spiritual person provided by a sample of Canadian university students and the data we analyzed came from three differing cultural samples (i.e., American, Indian, and Ugandan) of students, it seems as though existential well-being may not be a confound but rather an essential characteristic seen to be linked to higher levels of spirituality. If understood in this light, then the place of existential well-being within the content domain of spirituality may be reframed in terms of a directional relationship with the other dimensions. That is, existential well-being may be construed as an outcome variable concerning how one evaluates one’s own functioning as a product of the other spiritual dimensions.

In fact, this is something that has already been proposed. MacDonald [71] proffered a directional model with the ESI/ESI-R dimensions wherein Religiousness and the Experiential/Phenomenological Dimension comprise core social and biological factors that have a co-deterministic influence on the emergence and incorporation of cognitive schema into a person’s sense of self and reality (Cognitive Orientation and Paranormal Beliefs). In turn, he conjectured that all four of these dimensions have a bearing on how a person perceives and appraises his/her quality of life as manifested in existential well-being. Though not the focus of any empirical scrutiny to date, this bio-social-psychological model represents a promising development as it elevates the ESI/ESI-R dimensions beyond the mere description of spirituality to a level of organization, rigor, and explanatory power that fully integrates all of the dimensions in a compelling and scientifically testable way.

### Limitations

Notwithstanding the many key findings, the present study may be seen to suffer from a variety of limitations that need to be kept in mind when critically considering the meaning and generalizability of the results. First, all participants across all cultures were university students. While it may be argued that the consistent use of students served as a basis to make more apt comparisons across samples (e.g., it increased internal validity), the fact of the matter is that students differ from the general adult population in almost any country in terms of age, experience, and socioeconomic status [23, 103]. Resultingly, there is a strong need for research to be done across cultures with samples drawn from more diverse adult populations. Second, unevenness of sample sizes may be viewed as having a deleterious effect on the stability of our results, especially for those samples that are relatively small (i.e., Japanese and Slovakian). Future investigations replicating and extending our findings with larger samples are needed. Third, even though we included two measures of spirituality developed via different means in an effort to see if test construction strategy and test content had an impact on our results, both instruments are descriptive and not theory based. As well, our study lacked additional criterion measures that could have been used to better determine if the ESI-R and SAL produce similar patterns of associations across cultures. Behavioral rating variables such as frequency of attendance to religious events and/or frequency of engagement in private spiritual activities (e.g., prayer or meditation) and a broader array of health variables would be good to incorporate into any future cross-cultural studies as would a range of measures that are both descriptive and theory-driven. Fourth, despite evidence generally supporting the structural invariance of MacDonald’s [12] five dimensional model, our findings showed that the instrument demonstrated greater problems with goodness-of-fit with non-English language samples. While we attempted to ensure that translations were done adequately in terms of preservation of essential content and meaning of the items, we could have done more to better evaluate linguistic equivalence and cultural adaptedness prior to data gathering (e.g., we could have completed pilot testing of the translated ESI to identify potential problems with the translations) [104–106]. As a result, we cannot conclude with certainty that the relatively poorer fit of observed with non-English language samples was due to inadequacies with the translations or to bonafide cultural differences.

## Conclusions

Spirituality is an area of human functioning that has garnered greater attention and legitimatization and will undoubtedly continue to be the focus of research for many years to come. Nevertheless, the philosophical and methodological challenges it presents to science are substantial and should not be ignored. The present study serves to elevate our awareness of these complexities by highlighting the importance of culture and language in how spirituality is conceptualized, operationalized and measured. It is our sincere hope that the findings from this investigation contribute to more sensitive and socioculturally contextualized approach to theory development and inquiry.
